# Integrated Assessment of Coastal Exposure and Social Vulnerability to Coastal Hazards in East Africa

**DOI:** 10.1007/s12237-021-00930-5

**Published:** 2021-05-13

**Authors:** Caridad Ballesteros, Luciana S. Esteves

**Affiliations:** Department of Life & Environmental Sciences, Faculty of Science & Technology, Bournemouth University, Fern Barrow, Poole, BH12 5BB UK

**Keywords:** Coastal hazards, Exposure, Vulnerability, Index, Ecosystem-based management, East Africa

## Abstract

An index of vulnerability to coastal change, integrating indices of social vulnerability and exposure to coastal hazards, was created for East Africa to identify ‘areas of priority concern’ for risk reduction. Currently, 22% of East Africa’s coastline and 3.5 million people are at higher levels of exposure to coastal hazards, which would increase, respectively, to 39% and 6.9 million people if mangroves, coral reefs and seagrasses are lost. Madagascar and Mozambique show the largest proportion of the coastline at higher exposure, while Kenya and Tanzania benefit the most from natural coastal protection. Coral reefs protect 2.5 million people from higher exposure, mostly in Mombasa, Zanzibar and Dar es Salaam. Considering Mozambique, Kenya and Tanzania, the latter is the least, and the former is the most vulnerable. Under current conditions, 17 (out of 86) coastal districts are considered ‘areas of priority concern’; four of these are critically exposed as over 90% of their shoreline length are at higher exposure (Zavala, Inharrime, Manhiça and Mandlakaze, all in southern Mozambique). These locations are of critical concern for any present or future coastal development due to the high level of exposure posed to both vulnerable people and investments. Habitat loss would increase the number of ‘priority concern’ districts to 24; some would show great increase in the population exposed (e.g. Pemba and Mossuril in Mozambique). Applying this knowledge to identify where ecosystem-based management should be prioritised to promote social and environmental resilience is timely and urgent in East Africa.

**Supplementary Information:**

The online version contains supplementary material available at 10.1007/s12237-021-00930-5.

## Introduction

Natural hazards disproportionately affect the most impoverished and vulnerable people and are a key reason preventing poverty reduction (Hallegatte et al. 2017). Countries bordering the Western Indian Ocean are within the poorest in the world; Mozambique and Madagascar are particularly prone to cyclones and extreme weather events (e.g. Klinman and Reason 2008; Mavume et al. 2009; Fitchett and Grab 2014; Devi 2019). Mozambique, Madagascar and Tanzania are within the world’s top seven countries most susceptible to coastal hazards (Welle et al. 2014) where an increased number of people will be affected due to population growth and climate change (Neumann et al. 2015). The population in sub-Saharan Africa is projected to double (from one to over two billion) between 2019 and 2050 and become the world’s most populous region around 2062 (UN Department of Economic and Social Affairs 2019). In East Africa (EA), sea level rise and other climate change effects are expected to aggravate the severity of coastal hazards, which will affect more people more often (Abuodha 2009; Brown et al. 2009; Hinkel et al. 2012; Kebede and Nicholls 2012; Niang et al. 2014; Neumann et al. 2015).

The EA is one of the world’s regions most sensitive and vulnerable to climate change (Rakotobe 2012; Niang et al. 2014), with its effects projected (>90% likelihood) to halve the GDP per capita in Mozambique, Tanzania, Kenya and Madagascar by 2100 (Burke et al. 2015). Further, the region is experiencing fast and critical environmental and socioeconomic changes driven by foreign investment in large coastal infrastructure (e.g. port expansions in Lamu, Bagamoyo, Beira and Toamasina) and (before the COVID-19 pandemic) a growing tourism targeted to international visitors. These coastal areas are likely to see a faster and greater increase in population as people seek job opportunities and better living conditions (Neumann et al. 2015). The combination of rapid population growth and poor planning of new coastal developments leads to important socioeconomic and environmental changes, often compromising the preservation of coastal ecosystems (Wagner 2008) and disproportionately affecting the most vulnerable people, who depend the most on ecosystem services (Samoilys et al. 2015).

EA has rich coastal and marine ecosystems, including 5% of the world’s mangrove cover and a large diversity of coral reefs and seagrass beds (UNEP-Nairobi Convention and WIOMSA 2015), which support the livelihoods of many coastal communities heavily dependent on natural resources (Obura 2012; Rao et al. 2015; Samoilys et al. 2015; Ghermandi et al. 2019). These ecosystems play an important role in protecting the coast against storms as they dissipate wave energy and reduce flooding risk (Liquete et al. 2013; Spalding et al. 2014; Beck et al. 2018). However, despite the multiple ecosystem services coastal habitats provide, they are under threat from development pressures, climate change, limited protection and poor coastal management plans (UNEP-Nairobi Convention 2009; Dasgupta 2021).

Understanding the various components of risk is necessary to inform plans and effectively allocate resources to mitigate potential impacts (Oulahen et al. 2019). The use of indicators enables the categorisation and integration of multiple variables to define levels of exposure and vulnerability (Gallopín 1997; McLaughlin and Cooper 2010; Nguyen et al. 2016; Bevacqua et al. 2018). Since the pioneering coastal vulnerability index (Gornitz 1990) incorporating physical variables to assess exposure to sea level rise, many approaches have been developed (Bevacqua et al. 2018) using socioeconomic metrics (e.g. Cutter et al. 2003; Boruff et al. 2005; Ran et al. 2020) and integrating biophysical and social indicators (e.g. Arkema et al. 2013). The application of such indices is very limited in EA, often focusing on social vulnerability at the local or district level (Hahn et al. 2009; Zacarias 2019). Cabral et al. (2017) calculated an index of exposure to coastal hazards for Mozambique considering the effect of coastal habitats. This approach is expanded here to produce the first integrated assessment for the EA region (Mozambique, Madagascar, Tanzania and Kenya) that combines relative indices of exposure and social vulnerability to coastal hazards into an index of vulnerability to coastal change (IVCC).

Recognising that vulnerability is determined by the combination of physical, ecological and socioeconomic conditions that makes an individual, asset or systems more likely to be harmed by the impacts of hazards (UN General Assembly 2016; Bevacqua et al. 2018), here the terms exposure and vulnerability are used pragmatically to distinguish the biophysical and socioeconomic components of vulnerability. Therefore, the index of exposure (IE) assesses the degree to which an area is likely to be impacted by coastal hazards given its biophysical characteristics. The level of exposure is then transferred to the people (and assets) within the area. Social vulnerability refers to ‘sensitive populations that may be less likely to respond to, cope with, and recover from a natural disaster’ (Cutter and Finch 2008). Here, the social vulnerability index (SVI) combines key socioeconomic indicators to identify the populations in the study area likely to be more adversely affected by the impacts of coastal hazards (than others in the region) due to their less privileged conditions. The IVCC then provides a relative measure of coastal vulnerability incorporating biophysical and socioeconomic components.

This study uses, for the first time, the open source InVEST coastal vulnerability model (Sharp et al. 2020) in a supra-national assessment enabling comparison of relative levels of coastal exposure across Mozambique, Tanzania, Madagascar and Kenya. Previous studies had local (Hopper and Meixler 2016; Elliff and Kikuchi 2015), subnational (Jackson et al. 2020; Onat et al. 2018; Sajjad et al. 2020; Zhang et al. 2020) or national coverage (Arkema et al. 2013; Silver et al. 2019), including for Mozambique (Cabral et al. 2017). The results of this supra-national assessment are used to (a) identify the coastal provinces and districts most exposed and socially vulnerable to coastal hazards; (b) quantify the coastal population (within 5 km from the coastline) at higher levels of exposure; (c) assess the role of natural habitats in reducing exposure to coastal communities; and (d) discuss how the indices presented here can be used to inform decision-making aiming to reduce coastal vulnerability.

The key aspects of the work are summarised in the next four sections, with complementary information provided in Online Resources. The Methods describe how the IE and the SVI were calculated and aggregated into the IVCC. Online Resources detail data manipulation (ESM_1) and the statistical tests and data used in the validation of the IE and IVCC (ESM_2). The Results section addresses objectives a, b and c identified above. Data at the district and province levels are provided in Online Resources in tabular format (ESM_2). The Discussion addresses objective d considering applications of the results on three key aspects: nature-based risk reduction; build back better opportunities; and allocation of investments and international aid. The Conclusions summarise the key findings and highlight the contribution to knowledge brought by this study.

## Methods

### Index of Exposure to Coastal Hazards (IE)

The IE was calculated using the open source InVEST 3.8 coastal vulnerability model (Sharp et al. 2020), herein the InVEST model, with some adaptations. This model expands from the coastal vulnerability index (CVI) approach (Gornitz 1990), which has been widely applied in the last 30 years at a range of scales (e.g. Cooper and McLaughlin 1998; Hammar-Klose and Thieler 2001; Mujabar and Chandrasekar 2013; Ashraful Islam et al. 2016; Mahmood et al. 2020). The InVEST model has been increasingly used to assess the relative level of exposure to coastal hazards, as it enables integration of physical exposure, considering the effects of natural coastal protection offered by a range of habitats, and assessment of population affected (e.g. Arkema et al. 2013, 2017; Hopper and Meixler 2016; Onat et al. 2018; Silver et al. 2019; Sajjad et al. 2020; Zhang et al. 2020). To ensure data comparability across the EA region, the model was run using mostly freely available global data from a variety of sources (Table 1).

**Table 1:** Data input into the InVEST coastal vulnerability model for the calculation of the index of exposure (EI)

Data input	Variables	Sources
Administrative boundaries and coastline		Database of global administrative boundaries, GADM data 3.6 (2018), https://gadm.org
Relief	Topography (1 arc-second ~30m) Bathymetry (15 arc-second grid)	ASTER Global Digital Elevation Model, https://asterweb.jpl.nasa.gov General Bathymetric Chart of the Oceans (GEBCO_2019), https://www.gebco.net/data_and_products/gridded_bathymetry_data/gebco_2019/gebco_2019_info.html
Wind and wave exposure	Wind and wave data compiled from 8 years of WAVEWATCH III model hindcast reanalysis	Embedded in the InVEST model
Surge potential	Continental shelf	Embedded in the InVEST model 30-m depth contour line
Geomorphology	Shoreline change rates	Average annual rates for the period 1984–2016 at 500-m spacing along the coast (Luijendijk et al. 2018)
Habitats	Coral reefs	Global Distribution of Coral Reefs, http://data.unep-wcmc.org/datasets/1 (UNEP-WCMC et al. 2018)
	Mangroves	World Atlas of Mangroves, https://data.unep-wcmc.org/datasets/5 (Spalding et al. 2010)
	Seagrasses	Global Distribution of Seagrasses, http://data.unep-wcmc.org/datasets/7 (UNEP-WCMC, Short FT 2017)
Population	The Gridded Population of the World (GPWv4)	Center for International Earth Science Information Network (CIESIN)—Columbia University. 2018. NASA Socioeconomic Data and Applications Center (SEDAC). 10.7927/H4JW8BX5

The InVEST model allows the integration of seven variables: wave exposure, wind exposure, surge potential, relief, geomorphology, habitats and rates of sea level change. Each variable is ranked in five classes of exposure (Table 2) ranging from 1 (very low) to 5 (very high). The IE is calculated as the geometric mean of the variables’ ranking, as shown in Eq. 1, where R is the ranking of each variable.

1 $$ IE={\left({R}_{\mathrm{relief}}\ast {R}_{\mathrm{waves}}\ast {R}_{\mathrm{wind}}\ast {R}_{\mathrm{surge}}\ast {R}_{\mathrm{habitats}}\ast {R}_{\mathrm{shoreline}\ \mathrm{change}}\right)}^{1/6} $$

**Table 2:** Definition of classes and ranking values for each variable included in the index of exposure

Variables	Very low (1)	Low (2)	Moderate (3)	High (4)	Very high (5)
Relief (m)	12.00–233	8.00–12.00	4.00–8.00	2.00–4.00	0–2.00
Wave exposure^a^	0–0.75	0.75–3.00	3.00–18.70	18.75–48.00	48.01–219.77
Wind exposure	1st quantile	2nd quantile	3rd quantile	4th quantile	5th quantile
Surge potential	1st quantile	2nd quantile	3rd quantile	4th quantile	5th quantile
Natural habitats	Coral reef; mangrove	-	-	Seagrass	No habitat
Shoreline change rates (m/yr)	> + 2	+ 1 to + 2	−1 to +1	−2 to −1	< −2

^a^The maximum of the weighted average wave power of swells and seas as calculated by the InVEST model

For simplicity, the IE is analysed in this paper referring to three rather than five classes of relative exposure: lower (L), combining classes 1 and 2 (low and very low exposure); moderate (M); and higher (H), combining classes 4 and 5 (high and very high). Results are then summarised by presenting the average IE values and the proportion of the shoreline length classified as higher exposure for the different administrative levels (region, countries, provinces and districts). The term ‘provinces’ is generically used here to identify administrative ‘level 2’, which is the term used in Mozambique and equivalent to ‘counties’ in Kenya and ‘regions’ in Tanzania and Madagascar. Districts are the smallest administrative subdivision comparable across the countries (i.e. administrative ‘level 3’) for which socioeconomic data is available (see next section). Therefore, the analysis here is presented at district and province levels, although the IE component can be interrogated at finer resolutions.

The model parameterisation and data manipulation are described in Online Resource ESM_1. The method used here is similar to the one described by Silver et al. (2019) with the following modifications:

- Rather than creating and ranking the wave exposure and relief into five classes using quantiles, the class boundaries are customised to better reflect the likely differences in their contribution to exposure. Although class limits were arbitrarily defined (Table 2), this approach results in a more meaningful differentiation between classes, particularly for highly skewed distributions, such as wave energy and relief.
- As recommended in the model user guide (Sharp et al. 2020), shoreline change rates are used as the indicator for the susceptibility to erosion instead of the coastal typologies that represent the ‘geomorphology’ input. To our knowledge, this is the first study using this approach, which was facilitated by the publication of mean annual shoreline change rates for sandy shores worldwide (at 500 m spacing) estimated from satellite imagery for the period 1984–2016 (Luijendijk et al. 2018). Estimates are only available for sandy shores, and limitations related to the accuracy of estimates produced by non-supervised shoreline detection are recognised. However, these data offer a better proxy than a generalised coastal typology classification, which for this region is unavailable and would have to be extracted from satellite imagery at crude resolutions. The classes and ranking of shoreline change rates used in previous works (e.g. Gornitz et al. 1991; Nguyen et al. 2016) were adopted here (Table 2). The approach used to integrate the shoreline change rates into the model is described in Online Resource ESM_1.
- Rates of sea level change were not included in the analysis due to the scarcity of data (e.g. Cabral et al. 2017) that could be confidently applied to meaningfully differentiate levels of exposure across the region.
- The index is calculated using a spatial resolution of 1 km^2^, as done in previous studies for national and sub-national coverage (e.g. Arkema et al. 2013; Cabral et al. 2017; Sajjad et al. 2020). Although coarser than the 250-m^2^ resolution used by Silver et al. (2019), it is considered sufficient to identify the shoreline segments most exposed to coastal hazards within the region when comparing results at district and province levels. It is out of the scope of this study to identify localised effects not captured at this spatial resolution.

### Social Vulnerability Index (SVI)

Social vulnerability to natural hazards is most commonly measured through multiple indicators that reflect people’s limited access to resources, lower physical ability and marginalised groups due to their lower political or economic power, beliefs and gender (Cutter et al. 2003). Indicators of social vulnerability usually relate to ethnicity, gender, age (elderly and children), poverty/wealth (social class) and housing standards (Cutter and Finch 2008). The development of a SVI aggregating selected indicators is often the approach used to highlight spatial differences and identify the locations with the most vulnerable population (Ogie and Pradhan 2019). Most SVI approaches have been devised for national (e.g. Cutter et al. 2003) or subnational studies of data-rich (Ogie and Pradhan 2019) geographies, often with the objective of characterising place-based social vulnerabilities in addition to highlighting spatial differences. Similar studies applied in less data-rich contexts have identified the need to adjust the selection of indicators to suit the objective of the study and the realities of the study area (e.g. Hummell et al. 2016; Aksha et al. 2019).

The SVI here is used to support a comparative analysis of locations across countries, and the selection of indicators must consider the constraints related to the availability of comparable data. With the key objective of identifying districts in the region of relative higher vulnerability, the focus lies on geographical differences with comparable metrics available at this scale, and it is out of the scope here to provide a characterisation of local social vulnerabilities. Here, the SVI combines eight socioeconomic variables (Table 3) that are often used as indicators of social vulnerability to natural hazards, particularly the ones focusing on coastal areas in developing countries (e.g. Mazumdar and Paul 2016; Nguyen et al. 2019; Rabby et al. 2019; Sajjad and Chan 2019). The choice of variables used here (see Online Resource ESM_1 for details) considered data availability and comparability across the region of indicators that were deemed suitable to identify spatial differences in social vulnerability to coastal hazards within East Africa.

**Table 3:** Socioeconomic variables included in the SVI and sources of data

Data	Variables	Sources
Age	% of population < 4 years old % of population > 65 years old	Kenya National Bureau of Statistics (KNBS) and Society for International Development-East Africa (SID) (2013) Exploring Kenya’s Inequality. https://www.knbs.or.ke/?page_id=3142 Kenya National Bureau of Statistics (KNBS) (2015) County Statistical Abstract. https://www.knbs.or.ke/?page_id=3142 National Bureau of Statistics, Tanzania (NBS) (2016) Basic Demographic and Socio-Economic Profile. Population and Housing Census 2012. https://www.nbs.go.tz/index.php/en/regional-profiles Instituto Nacional de Estadística de Mozambique (INE) (2013) http://www.ine.gov.mz/estatisticas/estatisticas-territorias-distritais
Population growth and density	% average annual growth Inhabitants/km^2^	
Education	% illiteracy rate	
Housing standards	% of houses made of natural materials (floor, walls and roof)	
Sanitation	% population with unimproved water source % population unimproved human waste collection	

Note: Madagascar was excluded from the SVI as the census data (1993) were considered outdated and not comparable with the other countries

The indicators were based on household data obtained from the most recent national census data (Table 3) aggregated at the smallest administrative subdivision comparable across the countries (i.e. ‘level 3’ or districts). The SVI was calculated for coastal districts in Kenya, Tanzania and Mozambique. Madagascar was excluded as the most recent census data available dates from 1993 and was deemed outdated in comparison with the data available for the other countries (2012–2015). Kenya conducted a new census in 2019, but results at district level were not yet available at the time of writing. Following common practice to standardise variables of diverse measures, indicators were transformed using natural logarithm and normalised using *z*-scores (e.g. Tapsell et al. 2002; Mazumdar and Paul 2016). The *z*-scores were summed (linear aggregation) based on the direction of the indicator’s influence (Cutter and Finch 2008) and ranked from 1 (very low social vulnerability, first quantile) to 5 (very high social vulnerability, fifth quantile).

### Index of Vulnerability to Coastal Change (IVCC)

Recent reviews of the literature concerning vulnerability to natural hazards conclude that vulnerability is a concept intrinsic to both places and people and their biophysical and socioeconomic characteristics (e.g. Bevacqua et al. 2018; Ran et al. 2020). It is thus relevant to integrate biophysical exposure and social vulnerability to hazards to gain an overview of all contributing factors, particularly to prioritise risk management interventions (e.g. Mavromatidi et al. 2018). The IVCC brings together biophysical and socioeconomic metrics to offer a more comprehensive analysis of districts where both exposure and social vulnerability to coastal hazards are highest.

At district level, the IVCC is produced as the sum of the SVI and the average IE rankings of all data points within the district administrative boundary. Similarly, at province level, the IVCC is calculated as the sum of the average SVI of the province’s districts and the average IE rankings of all data points within the province boundaries. As the SVI was not calculated for Madagascar, the IVCC is also not available for this country. It is important to emphasise that the IVCC and its components (IE and SVI) are relative measures; any indication of low or high exposure should be considered solely in comparison to other coastal locations within the region studied here.

### Natural Habitat Scenarios

The InVEST model accounts for the effects of coastal habitats in reducing exposure to hazards at any shoreline segment that falls within a user-defined radius of relevant habitats. Differences in the protective capacity of each habitat (e.g. through dissipation of wave energy, attenuation of storm surges, retention of sediment, etc.) are considered through a relative ranking. In this study, the relative ranking (Table 2) and the user-defined radius (Online Resource ESM_1) were the same adopted by Arkema et al. (2013) and Sajjad et al. (2020). InVEST does not model the effects of habitats on hydrodynamics; therefore, the approach offered is a scaling factor informed by empirical studies rather than actual simulations.

To assess the role of coastal habitats in reducing coastal exposure, five scenarios were created considering the presence or absence of mangroves, coral reefs and seagrasses. In addition to the two scenarios that include or exclude all habitats (e.g. Arkema et al. 2013; Cabral et al. 2017; Silver et al. 2019; Sajjad et al. 2020), three other scenarios were created to assess the effect of ‘losing’ specific habitats. These scenarios where one or all habitats are completely lost are useful to assess where and how much they contribute to reduce exposure to coastal hazards; they are not intended to reflect future conditions (Silver et al. 2019). The number of people living in areas of high exposure to coastal hazards was calculated (using a 5-km buffer) for each scenario. The five scenarios are the following:

- *Scenario 1 (all habitats):* this scenario reflects the ‘current’ level of exposure, which accounts for the presence of the mangroves, coral reefs and seagrasses for which data are available for the four countries (Table 1).
- *Scenario 2 (no corals)*: the *R*_habitats_ is calculated considering the presence of mangroves and seagrasses only, to assess the effects caused by the loss of coral reefs.
- *Scenario 3 (no mangrove)*: only coral reefs and seagrasses are included in the *R*_habitats_ to evaluate the changes in coastal exposure resulting from the loss of mangroves.
- *Scenario 4 (no seagrasses)*: only coral reefs and mangroves are included in the *R*_habitats_ to assess the effects of seagrass habitats loss on the IE.
- *Scenario 5 (no habitats)*: this scenario considers the absence of all coastal habitats, which reflects the highest level of exposure from this indicator (*R*_habitats_ = 5).

### Model Validation

The InVEST model’s ability to reflect the levels of exposure to coastal hazards was successfully validated for Mozambique by Cabral et al. (2017); therefore, a similar approach was adopted here with some adaptations. These authors have tested for significant differences in the number of coastal hazard events and the events that have caused fatalities obtained from the DesInventar Database (UNISDR 2016) between ‘exposed’ and ‘less exposed’ coasts at ‘current conditions’ (i.e. scenario 1). They have classified as ‘exposed’ the coastal districts with ≥ 10% of the data points along their shoreline ranked as high or very high IE by the InVEST model. As the threshold of 10% can be considered very low to adequately distinguish between ‘exposed’ and ‘less exposed’ coasts, here the validation was tested using 20%, 25%, 30% and 50% of the data points as the threshold.

The analysis was conducted at coastal district level, except for Kenya, as the DesInventar data for this country are provided at county level. Two DesInventar variables were used in the validation, the number of ‘coastal’ hazard events (i.e. tropical depressions, surges, strong winds, storms, heavy rains, floods, flash floods and cyclones) and the number of events causing fatalities. The validation data can be found in Online Resource ESM_2. Non-parametric tests were used as the data in all sets were skewed and did not follow a normal distribution. All statistical analyses were undertaken using IBM SPSS Statistics 26.

The Mann-Whitney U test showed significant differences (95% confidence interval) in both the number of events and the number of events causing fatalities between ‘exposed’ and ‘less exposed’ defined by all four thresholds (all results are presented in Online Resource ESM_2). Spearman’s correlation test showed a significant positive correlation between the % of shoreline length at higher exposure and the number of events (rho=0.321, *p*<0.000, *N*=120) and the number of events causing fatalities (rho=0.408, *p*<0.000, *N*=120). These results demonstrate that the InVEST model IE provides a reasonable estimate of relative exposure to coastal hazards and that the % of shoreline length at higher exposure (using thresholds varying from 20 to 50%) is a suitable proxy to be used for comparative analysis at district level.

Similarly, the suitability of the IVCC was ascertained by the significant positive correlations found for both the number of events (rho=0.647, *p*<0.000, *N*=75) and the number of events causing fatalities (rho=0.497, *p*<0.000, *N*=75) reported for each district (county in Kenya). Additionally, the Mann-Whitney U tests showed significantly higher IVCC values (ranked median) in ‘exposed’ than in ‘less exposed’ districts at 99% confidence interval for all four threshold classifications (e.g. *U*=143, *p*=0.005 for threshold of 50% shoreline length at higher exposure). Based on these results, the IVCC was considered an appropriate proxy of social vulnerability to coastal change.

## Results

About 22% of the 22,112 km of the EA shoreline length analysed here show higher levels of exposure to coastal hazards (Table 4). Mozambique and Madagascar are the countries with the largest proportion of their coastline length at higher levels of exposure (23.4% and 25.6%, respectively); Tanzania and Kenya have 14.3% and 9.8%, respectively (Table 4). The average IE values and proportion of the coastline length at higher exposure for each country and their respective provinces and districts are presented in Online Resource ESM_2.

**Table 4:** Coastline length and population at higher exposure for scenarios 1 (all habitats) and 5 (no habitats) in East Africa, per country and their respective most exposed provinces (the highest % of coastline length at higher exposure in scenario 1)

	Total coastline length (km)	Coastline length (km) at higher exposure		Coastline length (%) at higher exposure		Population (10^3^) at higher exposure (within 5 km)	
		With habitats	No habitats	With habitats	No habitats	With habitats	No habitats
East Africa	22,112	4,827	8,603	21.8	38.9	3,536.8	6,898.8
Kenya	1,591	228	581	14.3	36.5	314.6	1,298.5
Tana River	99	64	76	64.6	76.8	15.2	15.6
Tanzania	3,138	309	1,039	9.8	33.1	405.1	2,204.7
Pwani	636	159	339	25.0	53.3	53.6	80.8
Mozambique	7,146	1,670	2,500	23.4	34.9	1,660.4	1,899.9
Gaza	202	154	154	76.2	76.2	76.3	76.3
Madagascar	10,237	2,620	4,483	25.6	43.8	1,156.8	1,495.7
Androy	211	172	187	81.5	88.6	71.8	72.1

**Fig. 1:**
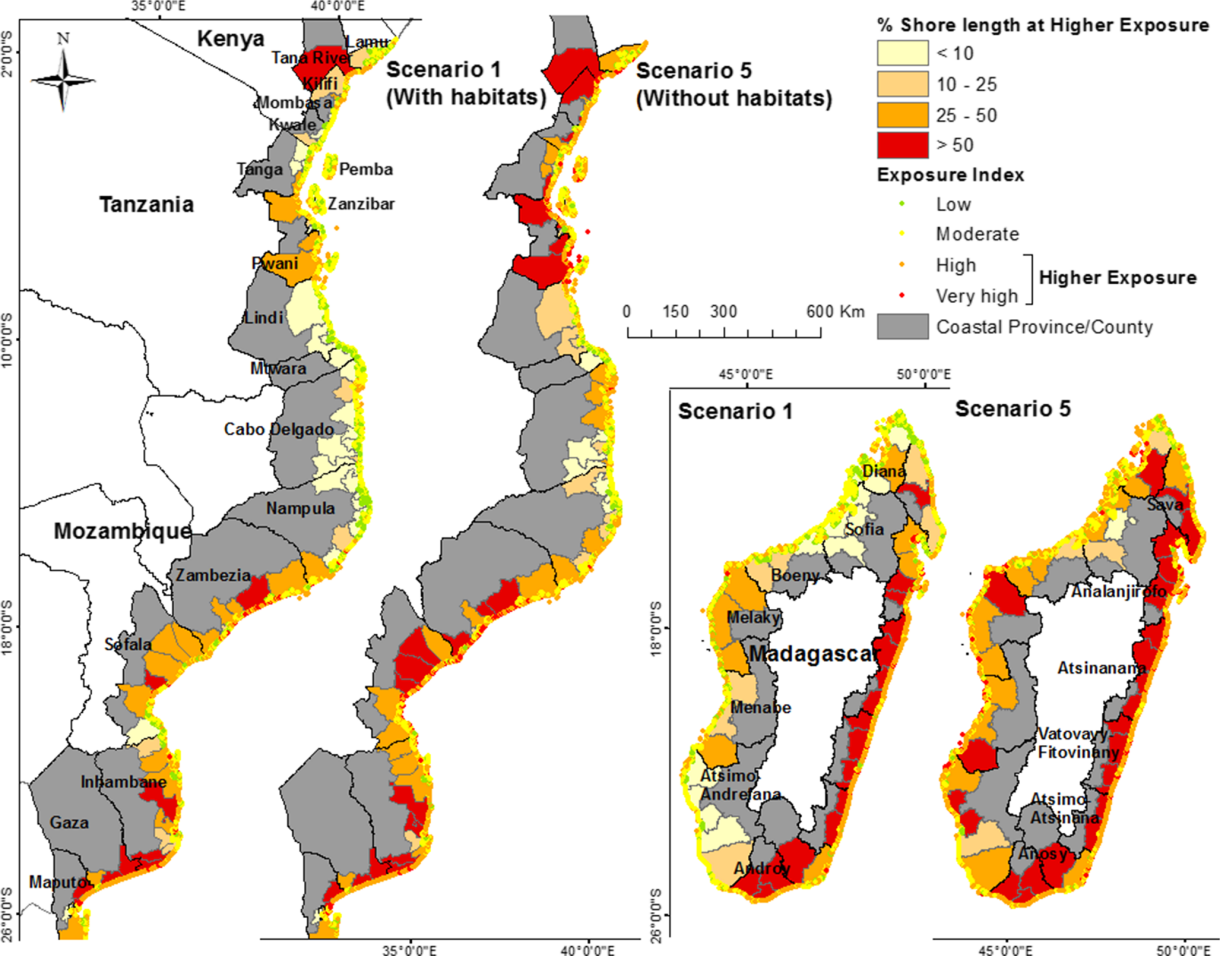
Exposure index ranking (data points at 1-km distancing along the coastline) and proportion of district shoreline length at higher exposure for habitat scenarios 1 and 5

There are large variations in the distribution of coastal stretches at higher exposure (Fig. 1). At district level, the proportion of coastline length at higher exposure ranges from 0 (in all countries) to 100% in Mozambique (Zavala, Mandlakazi and Manhiça) and Madagascar (Ampasimanolotra and Farafangana), 65% in Kenya (Garsen) and 47% in Tanzania (Rufiji). The 27 (out of 131) ‘exposed’ coastal districts (>50% of coastline at higher exposure) are located mainly in eastern Madagascar and southern Mozambique; only one is found in Kenya and none in Tanzania (Fig. 1). At province level, the range varies from 0 (Mombasa, Kenya, and Mtwara; Zanzibar North and Zanzibar South; and Central Tanzania) to 81.5% (Androy, Madagascar).

Natural habitats play a key role in reducing the level of exposure to coastal hazards in EA. Without habitats (scenario 5), the coastal length at higher exposure in the region will almost double, from 4,827 to 8,603 km (Table 4). A further 23 coastal districts (to a total of 50) would be classified as ‘exposed’ (see Online Resource ESM_2). Tanzania and Kenya benefit the most from natural coastal protection. If coastal habitats are lost, they would see a greater increase in the proportion of their coastline at higher exposure, from 9.8 to 33.1% in Tanzania and from 14.3 to 36.5% in Kenya (Table 4). In scenario 5, Kenya would have the second largest proportion of shoreline length at higher exposure, behind Madagascar only. Madagascar would have the largest increase in absolute coastline length, with additional 1853 km (18.2% of the total shoreline length) classified at higher exposure levels.

The provinces of Gaza (Mozambique) and Androy (Madagascar) are the most exposed in EA (Table 4). They have high wave and wind exposure and receive little natural protection from coastal habitats (i.e. no difference in coastal length at higher exposure between habitat scenarios). In other provinces, the loss of coastal habitats (scenario 5) would increase the proportion of the coast at higher exposure by as much as eight times. The largest increases among provinces are found in Sofia (Madagascar) from 3.8 to 30.8%, Dar es Salaam (Tanzania) from 4.9 to 35.7% and Kwale (Kenya) from 7 to 43%. Dramatic increases are observed at district level, particularly in Kenya, notably in Kilifi South (from 0 to 92%), Matuga (from 0 to 77%) and Nyali (from 0 to 57%).

**Fig. 2:**
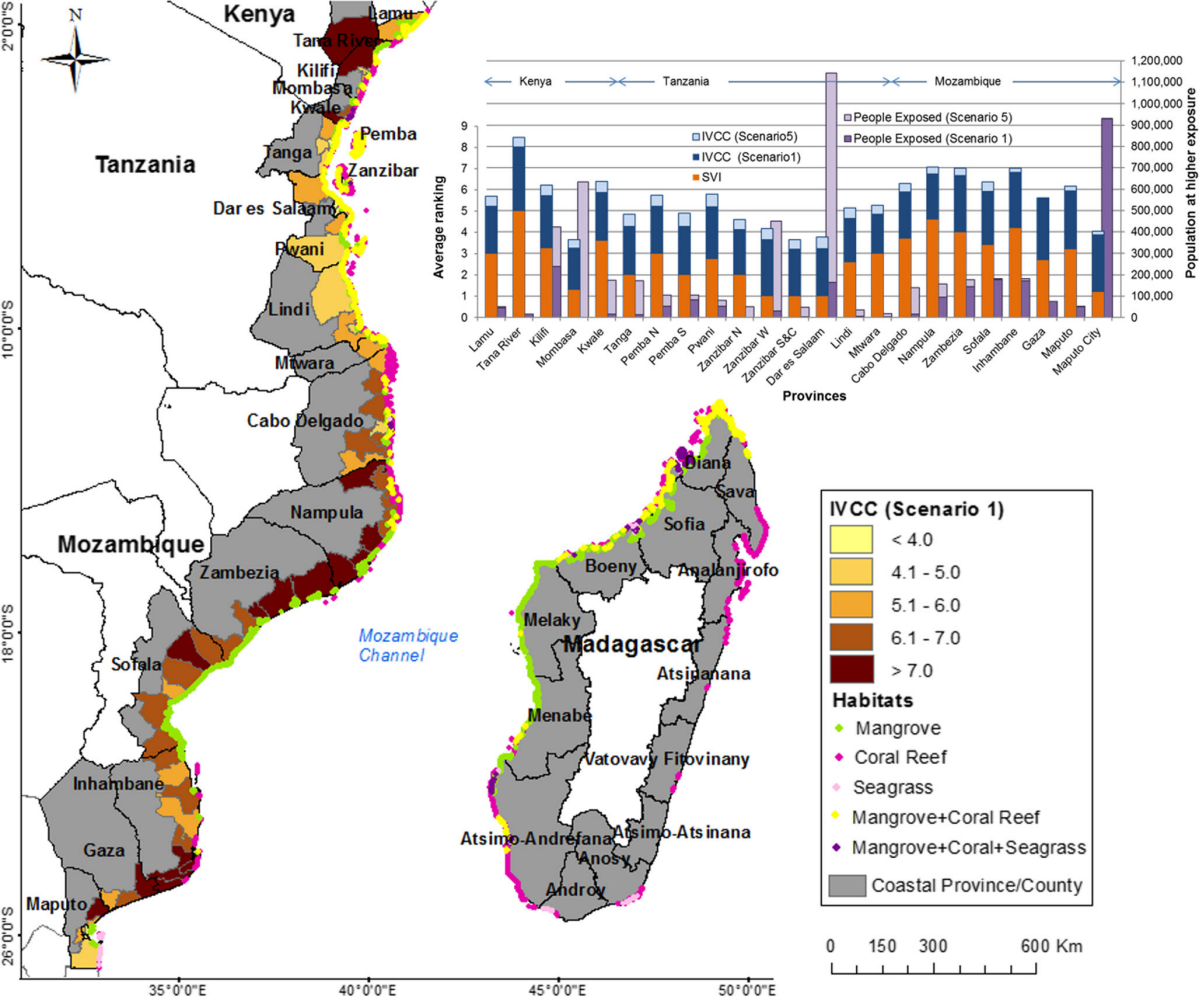
Spatial distribution of coastal habitats and the index of vulnerability to coastal change (IVCC) value for districts. The insert graph shows the IVCC value and the population at higher exposure (within 5 km of the coastline) for scenarios 1 and 5 and the social vulnerability index (SVI) for provinces. The coastal exposure index for each province for scenarios 1 and 5 can be inferred from the difference between the IVCC and SVI. Madagascar was excluded from the calculations of SVI and IVCC as the available census data are outdated in relation to the other countries

In scenario 5, almost 6.9 million people in EA would be within 5 km from a coastline ranked at higher exposure (Table 4), an increase of 3.3 million from current conditions (scenario 1); 54% (1.8 million) in Tanzania, 29% (1 million) in Kenya and the rest in Mozambique and Madagascar. The relative importance of specific habitats in providing coastal protection is geographically and scale dependent. At the regional scale, mangroves contribute to coastal protection along about 60% of the coastline and coral reefs along 34% (both coexisting along 18%). However, coral reefs protect a higher number of people (about 2.5 million) from higher exposure levels, and they have a greater effect in reducing the level of exposure (i.e. a higher number of coastal locations will move to a higher IE ranking in scenario 2 than in scenario 3). In southwestern Madagascar, northern Mozambique, Tanzania and Kenya, both mangroves and coral reefs offer important natural coastal protection (Fig. 2), although one or the other may dominate at district and local levels. Mangroves are the habitats contributing the most to reduce exposure in central Mozambique and western Madagascar. Seagrasses contribute to natural coastal protection along 1810 km (8%) of EA’s coastlines and are particularly important in southern Mozambique and southern Madagascar.

There are six districts with more than 100,000 people within 5 km of coastlines at higher exposure in scenario 1: Maputo (930,000) and Dondo (150,000, mostly in the city of Beira) in Mozambique; Toamasina Rural (165,000) and Farafangana (105,000) in the east coast of Madagascar; Malindi (110,000) in Kenya; and Kinondoni (160,000) in Dar es Salaam, Tanzania. Except the latter, these areas receive little natural protection from coastal habitats; therefore, little change in the population at higher exposure is expected in scenarios of habitat loss. In Kinondoni, the population at higher exposure would increase to 573,000 in scenario 2 and 780,000 in scenario 5; coral reefs alone protect over 400,000 people from higher exposure. In scenario 5, the number of districts with more than 100,000 people at higher exposure would treble, the same two in Mozambique and increasing to three in Madagascar, six in Tanzania and seven in Kenya. Great increases in population at higher exposure would occur in the largest urban centres in Dar es Salaam from 163,000 to 1.1 million, Zanzibar Town (Tanzania) from 31,000 to 450,000 and Mombasa from zero to 634,000 (in Kenya). In these three locations alone, coral reefs protect 1.8 million people, preventing about 95% of the increase in population at higher exposure in Mombasa and Zanzibar Town and 63% in Dar es Salaam.

### Index of Vulnerability to Coastal Change

Of the three countries analysed here, Mozambique shows the highest average SVI (3.5), followed by Kenya (2.6) and Tanzania (2.0). Mozambique also shows a higher average IE (2.5) than the other two countries (2.3 for both) in scenario 1, and all countries have the same average IE (2.8) in scenario 5. Therefore, Mozambique shows the highest average IVCC in all scenarios (ranging from 6.0 to 6.3).

In comparative terms, social vulnerability is considerably lower in Tanzania, where the highest average is SVI=3 at province level (in Pemba North and Mtwara). In contrast, only four provinces have SVI≤3 in the other countries, Maputo City and Gaza in Mozambique and Mombasa and Lamu in Kenya. Pemba North and Pwani are the provinces with the highest IVCC in Tanzania (both ranging from 5.2 in scenario 1 to 5.8 in scenario 5). Four provinces have SVI≥4, Tana River in Kenya (SVI=5), Nampula (SVI=4.6), Inhambane (SVI=4.2) and Zambezia (SVI=4.0) in Mozambique; these provinces also show the highest IVCC (Fig. 2). Tana River (Kenya) is the province with highest IVCC in all scenarios (ranging from 8.1 in scenario 1 to 8.5 in scenario 5), followed by Inhambane (IVCC ranging from 6.9 to 7.0) and Nampula (IVCC ranging from 6.8 to 7.1). Illiteracy rates, sanitation levels, proportion of population of young children (aged <4 years) and poor housing standards are the factors contributing the most to these higher levels of social vulnerability. Nampula and Zambezia show the first and third highest levels of illiteracy in coastal EA, with Tana River in fourth position in EA and the highest in coastal Kenya. These provinces also show the highest percentage of houses built with natural materials (> 91%) when compared to the average in coastal EA (70% for all provinces). Nampula and Zambezia also show the highest proportion of the population lacking access to improved water supply (71% and 65%, respectively; average for coastal EA 40%) and human waste collection (93% and 92%, respectively; average for coastal EA 58%).

Districts with an IVCC ranking >7 are considered here ‘areas of priority concern’, as this value reflects that both the SVI and IE are moderately high or that at least one is ranked highest. Seventeen (out of 86) districts show IVCC>7 in scenario 1, three in Kenya and 14 in Mozambique (Fig. 2), the latter increasing to 21 in scenario 5 and 24 in total. Four of these districts in Mozambique (Zavala, Inharrime, Manhiça and Mandlakaze) show >90% of their coastline at higher exposure in scenario 1, placing them as ‘critical areas’ for any present or future coastal development. Of these four districts, Zavala (Inhambane province) shows the largest population at higher exposure (25,000) and also the highest IVCC (8.75) in EA, thus leading the undesired position of most critical area of priority concern. The highest IVCC in Kenya and Tanzania is in Garsen (Tana River, ranging from 8.05 to 8.48) and Micheweni (Pemba North, ranging from 6.24 to 6.73), respectively. It is important to note that the largest populations at higher exposure are found in districts showing lower SVI and IVCC, as observed in Dar es Salaam, Maputo City, Mombasa and Zanzibar West (Fig. 2). These large urban centres have a larger proportion of the population benefiting from improved sanitation and housing standards than in rural areas, which is reflected in the indicators forming the SVI. The SVI calculated at the district level does not capture the large inequalities that exist within its boundaries.

## Discussion

This research pioneers for producing the first integrated assessment of social vulnerability and exposure to coastal hazards covering the EA region and for evaluating the natural protection offered by coastal habitats across four countries. Integrating biophysical and social indicators provides a more comprehensive reflection of vulnerability required to inform planning and decision-making (Bevacqua et al. 2018; Onat et al. 2018; Maanan et al. 2018). Madagascar and Mozambique are more exposed to coastal hazards and have a larger population at higher exposure than Kenya and Tanzania. Indeed, multiple global analyses show Mozambique and Madagascar within the world’s most vulnerable countries to weather and climate-related hazards (Dasgupta et al. 2009). However, if coastal habitats are lost, Kenya would surpass Mozambique in proportion of the shoreline length at higher exposure, and Tanzania would have the highest number of people at higher exposure.

By covering a large region, this integrated assessment produced layers of information that can be interrogated at a range of spatial scales to identify areas of priority concern or warranting further investigation (Silver et al. 2019; Jackson et al. 2020) and the factors contributing the most to elevate exposure or social vulnerability. Using this knowledge to inform decision-making is timely and urgent in EA (at all administrative levels), where fast social and environmental changes are occurring and may inadvertently increase biophysical and/or social vulnerabilities (e.g. coastal habitat loss reducing natural protection in socially vulnerable communities). It is therefore important to discuss how the IE, SVI and IVCC data can be applied to inform planning and risk reduction measures while recognising the limitations of the method (see next section on Limitations). Although focusing on EA, the applications discussed here are relevant to other developing regions or countries worldwide.

It is often claimed that coastal vulnerability indices are useful to inform disaster risk reduction (Onat et al. 2018; Sajjad et al. 2020), coastal planning and management (Martins et al. 2012; Serafim et al. 2019; Jackson et al. 2020; Sajjad et al. 2020), climate change adaptation (e.g. Onat et al. 2018; Zhang et al. 2020) and, when the InVEST model has been used, habitat conservation for coastal protection (Arkema et al. 2013; Cabral et al. 2017; Hopper and Meixler 2016; Silver et al. 2019; Sajjad et al. 2020). Some studies analyse specific socioeconomic aspects, such as natural protection to property value (Arkema et al. 2013; Jackson et al. 2020), land ownership (Hopper and Meixler 2016), population (Arkema et al. 2013; Jackson et al. 2020; Sajjad et al. 2020) and vulnerable people (Silver et al. 2019; Zhang et al. 2020). However, very few papers discuss how the results can be applied in practice, and only one describes an actual application. Results from Silver et al. (2019) informed government decisions in the Bahamas related to post-disaster recovery in the aftermath of hurricanes Joaquin (2015) and Matthew (2016) and led to a programme of mangrove restoration funded by the Inter-American Development Bank aiming to increase coastal resilience.

To reduce this knowledge gap, potential applications of the data produced here to inform decisions across administrative levels will be discussed focusing on allocation of investments and international aid; ecosystem-based risk reduction; and ‘building back better’. Building back better in recovery and reconstruction post-disaster is one of the priorities of the Sendai Framework for Disaster Risk Reduction (UNISDR 2015). The four countries analysed here are categorised as lower middle income (Kenya) and least developed countries (Mozambique, Tanzania and Madagascar) and included in the Development Assistance Committee (DAC) list of Official Development Assistance (ODA) recipients. Therefore, they are major recipients of international aid. In 2017, Tanzania, Kenya and Mozambique were ranked 3rd (USD 2.584 billion), 4th (USD 2.475 billion) and 9th (USD 1.776 billion) top ODA recipients of all aid to Africa (OECD 2019). As demonstrated by Silver et al. (2019), the data resulting from studies similar to this one can be used to guide international aid to areas most in need of risk reduction and identify locations where ecosystem-based coastal protection is particularly important and where both can benefit the most vulnerable people.

Large port expansions are ongoing in EA (e.g. Lamu, Bagamoyo, Toamasina, Beira), which strategically improve trade across and beyond the region and significantly contribute to the national and local economies. Understanding exposure at port locations is essential (Mutombo and Ölçer 2017), as high exposure can compromise the sustainability of operations in the longer term and jeopardise the intended economic benefits (Becker et al. 2012; Nursey-Bray et al. 2013). Our data show that Toamasina (east Madagascar) shows high exposure to hazards, mainly due to waves and winds, the top two ‘climate’ concerns for ports (Mutombo and Ölçer 2017). Loss of coral reefs would make this a critical area of priority concern. The district of Bagamoyo (Tanzania) shows moderate SVI and IE and will become exposed if mangroves and coral reefs are lost, which can raise the IVCC from 5.7 (scenario 1) to 6.4 (scenario 5). This increase does not account for changes in socioeconomic conditions, and it is important that local development is able to foster improvements in the local communities, particularly for the most vulnerable people. Bagamoyo, for example, has a relatively higher proportion of elderly people than other coastal districts in the region (Table S10 in ESM_3). Inflow of young skilled employers from outside the local area might change the local demographics and reflect an ‘improvement’ in the social vulnerability metrics. On the other hand, changes in employment opportunities and access to natural resources can be detrimental to disadvantaged groups (e.g. the elderly), leading to their marginalisation and/or displacement. Identifying and resolving such issues require a better understanding of social vulnerabilities at the local level.

Coral reefs around Toamasina and Bagamoyo are already at very high risk from local threats (Burke et al. 2011), and reducing these threats would be important to ensure corals continue to offer natural coastal protection. These ports need to consider the sustainability of further expansions and incorporate adaptation plans to manage increased exposure due to both degradation of coral reefs and climate change. Similarly, these plans should consider opportunities to improve conditions of local communities to reduce social vulnerabilities, e.g. education and training opportunities, sanitation and better housing standards. Beira is the second largest port in Mozambique and currently benefits from substantial rail and road upgrades connecting the export route for the landlocked countries of Zimbabwe, Zambia and Malawi. Beira is already experiencing high exposure, as evidenced by the destruction of port buildings and disruption to transport routes for months in the aftermath of cyclone Idai (March 2019), which affected 1.8 million people, causing 600 deaths and 177,800 people displaced in Mozambique (Devi 2019).

The data can also be interrogated to assess the relative vulnerability to coastal change of other assets of national and international relevance, such as UNESCO World Heritage Sites. Vulnerability assessments and frameworks are emerging to evaluate the impact of hazards and climate change to different cultural assets (Daly 2014; Phillips 2015; Reeder-Myers 2015; Forino et al. 2016; Romão et al. 2016; Vojinovic et al. 2016). However, these frameworks are often qualitative and site-specific, which limits comparability and replication across sites. Such assessments focusing on cultural heritage are largely scarce in Africa (Fatorić and Seekamp 2017). A preliminary assessment indicates that UNESCO World Cultural Heritage Sites in coastal EA are in areas of low to moderate exposure. Mozambique Island is the only site in a district showing very high social vulnerability and likely to become ‘priority concern’ if corals are lost, and they are already at *very high risk* from local threats (Burke et al. 2011). In Zanzibar Town, loss of coral reefs would place 20% of its coastline and over 240,000 people at higher exposure, likely to heighten threats to the integrity of historic buildings. The regional assessment can identify which sites are at relative higher exposure and the key contributing factors, which can then be used, by UNESCO or the organisations managing the sites, to target more detailed investigations.

Results presented here show that conservation of coral reefs, mangroves and seagrasses would prevent 3.4 million people and over 3,700 km of EA’s coastline from higher levels of exposure to coastal hazards. It becomes evident that, along these coastlines, conservation of coastal habitats should be the primary risk reduction strategy (Francis et al. 2002). Analyses of habitat scenarios data identified the areas where each habitat offers greatest risk reduction and protection to people (Jackson et al. 2020). The district of Kilifi South (Kenya), for example, shows an increase from not having areas at high exposure in scenario 1 to 92% of the coastline (and over 95,000 people) at higher exposure in scenario 2, showing the importance of coral reefs at this location. Extending the Mombasa Marine National Reserve further north or creating a new reserve to protect and improve the health of coral reefs along Kilifi South could preserve valuable natural coastal protection benefiting a population of moderate SVI. The creation of marine reserves can help the conservation of habitats (Samoilys et al. 2015) and poverty alleviation (Wells et al. 2007). Poverty has been identified as a cause of ecosystems degradation (Wagner 2008; UNEP-Nairobi Convention and WIOMSA 2015). However, a bottom-up approach integrating local communities is necessary for ecosystem-based management to be effective (e.g. Kairo et al. 2001; Obura et al. 2002; Wagner 2005; Massuanganhe et al. 2015).

As priority concern districts already show high levels of exposure and little natural protection, they would benefit from habitat conservation where they exist, restoration of habitat where possible and maximising the use of green infrastructure. The benefits of coastal green infrastructure in reducing the impacts of coastal hazards have been demonstrated in developing (e.g. Silva et al. 2017) and developed countries (e.g. Ruckelshaus et al. 2016). International aid projects have identified mangrove restoration and green urban infrastructure to reduce exposure in Beira and other cities in Mozambique, but implementation so far has been limited (CES 2020). As demonstrated by Silver et al. (2019), IE data can be used to guide investments in habitat restoration. Mangrove deforestation is widespread in Mozambique (Macamo et al. 2016), and projects of environmental education to raise awareness about ecosystem services are needed to engage communities and stakeholders in conservation (Silver et al. 2019; Charrua et al. 2020). Mangroves require shelter from wave action to develop (e.g. Wagner 2004; Stewart and Fairfull 2008; McIvor et al. 2012), and opportunities for habitat restoration may be limited in very exposed areas. A recent study shows high potential for mangrove development in the areas of Beira and Maputo (Charrua et al. 2020), where nature-based solutions could increase coastal resilience benefiting large populations and the local and national economy.

This combination of nature-based approaches should also be considered to improve resilience in large urban centres (Losada et al. 2017; Mabula et al. 2017), such as Dar es Salaam, Zanzibar Town and Mombasa where coral reefs at very high risk from local threats reduce exposure to 1.8 million people (even though these areas show only a fraction of their coastline at higher exposure). Following the examples from mangrove restoration in the Bahamas (Silver et al. 2019), detailed numerical modelling can help identify the suitability of specific locations and the size of the area required to maximise coastal protection benefits. Similarly, fine-resolution studies are needed to understand local variations in the components of social vulnerability and develop place-based solutions accordingly (e.g. Cutter and Finch 2008).

Nature-based solutions are recognised as resource-efficient and sustainable approach to disaster risk reduction (Faivre et al. 2018) and increasingly applied in practice (Cohen-Shacham et al. 2016; Renaud et al. 2016; Fernandino et al. 2018), including in building back better strategies (CES 2020). Building back better creates greater benefits to communities and countries most hit by disasters due to reduction in damages resulting from more resilient structures and infrastructure built in less prone areas (Hallegatte et al. 2018). In exposed districts, coastal resilience depends on adequate planning for housing and critical infrastructure to avoid increasing the number of people and assets at risk and reduce the impacts from coastal hazards. In critically exposed districts, avoiding areas of high exposure becomes very challenging, as these are the great majority if not all the coastline. In these districts, build back better opportunities may require displacement to inland areas or to less exposed districts. Any new development or relocation must be carefully planned to reduce risks to critical infrastructure and create the maximum benefit to the most vulnerable people, so inequalities and costs from disasters are reduced (Hallegatte et al. 2018).

Population growth and development pressures pose a challenge for the management of coastal resources (Mbonile and Kivelia 2008; Sabai 2017) and can force more communities into exposed areas (Hallegatte et al. 2018). Population growth in least developed countries is 2.5 times faster than in the rest of the world, and there is a high probability that population will more than double between 2019 and 2050 in Tanzania, Mozambique and Madagascar (UN Department of Economic and Social Affairs 2019). Projections indicate a 50% increase in the number of babies in the next 30 years in relation to the last three decades in sub-Saharan Africa, including an increased rate from adolescent mothers in least developed countries, increasing the vulnerable population. Efforts to promote coastal resilience must aim to reduce both exposure and social vulnerability.

## Limitations

The integration of indicators into an index can help to synthesise complexity into simple terms, permitting comparisons across space and/or time (Gallopín 1997; Schneiderbauer and Ehrlich 2004; Vincent 2004). In this work, the integration of the indices into the IVCC enables a more comprehensive assessment of physical and socioeconomic conditions to identify areas of relatively higher coastal vulnerability within EA. Results identify priority areas where more detailed assessment is required for devising and implementing disaster risk reduction and coastal management measures at the local level. The IE data presented here allows downscaling to the local level to identify the variables influencing the levels of biophysical exposure. The same level of detail is not available for the components of social vulnerability due to constraints of data availability.

The SVI represents both hazard-dependent variables (e.g. house materials and sanitation) and hazard-independent variables, such as age, illiteracy, population growth and density (Schneiderbauer and Ehrlich 2004). Investment in structural resources to improve hazard-dependent variables, such as the material of houses and sanitation systems, can help to lessen the impacts of cyclones and floods, so reducing vulnerability levels to coastal hazards (e.g. Mazumdar and Paul 2016; Zacarias 2019). Here, the SVI was used as a means to identify large differences between districts and provinces and provide the socioeconomic component to the IVCC. For example, communities with higher literacy rates are expected to be better informed and more able to take precautionary measures pre- and post-disaster (Mazumdar and Paul 2016). For management purposes, it is important to understand how the measures and policies are likely to affect parameters on the ground, at finer resolution.

These results must be considered with caution, as they only reflect how locations compare in relation to others within the region of assessment. Therefore, areas ranked here as having low exposure or vulnerability may still be more exposed and vulnerable than other areas outside EA or show a different ranking if the area of assessment changes. Further, the SVI and IVCC exclude Madagascar (due to outdated data), and it is possible that this country would show high vulnerability levels. Madagascar is one of the poorest countries in the world ranked 162 out of 189 in the 2019 human development index (UNDP 2020).

The representativeness of the indices is largely dependent on data quality, and an assessment with regional coverage involves compromises related to temporality and spatial resolution. Here, the results are assumed to reflect contemporary conditions and offer no insights on changes through time. Availability of global data enabled IE calculations for EA, where data scarcity and sparse coverage are a problem. The datasets used here (Table 1) are the best available for regional comparability and suitable for this purpose but are less robust at local scales. The SVI was calculated based on census data, and differences exist in the way data are collected and reported in each country. This is the first time that indicators have been collated and analysed at district level, the smallest scale available from census data for the countries in this study. This scale of analysis is comparable to other similar studies of social vulnerability undertaken at national level in data-rich countries, such as in the USA (Cutter and Finch 2008), and suitable to identify districts where conditions are of greater concern. Most indicators reflect the percentage of the population in each district possessing the particular characteristic. Therefore, the indices identify the districts that have the highest aggregated value, and this may indeed obscure locations where a particular condition is of critical concern.

More detailed quantitative and qualitative analyses at the local level would be required to measure the social vulnerability of communities and to ensure effective adaptation at the household and community levels (e.g. Hahn et al. 2009; Zacarias 2019). This could also benefit the identification of the main indicators necessary to assess vulnerability in urban areas where social realities in EA can divert from rural areas. There are limitations with the aggregation of indicators, creating more opportunities for subjectivity, so these need to be validated (Vincent 2004). Validation of the IE and IVCC with empirical data at the district level (Online Resource ESM_2) gives confidence in the rankings presented here, but limitations associated with the selection of indicators and data quality must be taken into consideration to support management decisions at the local level. Analysis of exposure and vulnerability at any specific location requires data of higher resolution, investigation of local threats and, where relevant, support from detailed numerical modelling (e.g. Silver et al. 2019).

Limitations associated with the simplifications and assumptions of the InVEST model are well-described in the model documentation (Sharp et al. 2020), including that it lacks considerations of nearshore effects on waves and surge described by Sajjad et al. (2020). Despite recognised model limitations, the InVEST offers a simple and yet robust framework to assess relative exposure to coastal hazards that provides useful information for decision-makers even in data-poor areas (Silver et al. 2019).

It is worth drawing attention that the model uses an empirical formula that approximates the distance of ‘protection’ offered by different habitats or multiple habitats rather than actual process-based modelling. Additionally, it does not take into consideration the state of conservation of habitats (Cabral et al. 2017; Jackson et al. 2020), which is known to affect their ability to provide ecosystem services, including coastal protection (e.g. Danielsen et al. 2005; Spalding et al. 2014). Therefore, the level of natural protection may be overestimated where habitats are degraded. Jackson et al. (2020) adjusted the IE ranking produced by InVEST by ±0.5 to represent the effects of degraded or improved habitats. Applying an arbitrary adjustment value is a simple approach to avoid the ‘all or no’ coastal protection associated with habitat scenarios particularly for localised assessments where habitats are more homogenous. The addition of empirical formula reflecting the level of attenuation and the protective distance for altered habitats would be a welcome improvement to the model.

## Conclusions

This research pioneers for producing the first integrated assessment of social vulnerability and exposure to coastal hazards for East Africa. Combining levels of coastal exposure and social vulnerability with estimates of the population affected and the role of natural habitats in reducing exposure has brought an improved understanding of where these elements converge contributing to higher levels of vulnerability to coastal change. Results show that around 22% of the region’s coastline show high levels of exposure to coastal hazards and 3.5 million people live within 5 km of these coastlines. Coral reefs, mangroves and seagrasses prevent these figures from rising to 39% of the coastal length (an increase of over 3,770 km) and 6.9 million people. Currently, Mozambique and Madagascar are the countries most exposed to coastal hazards. However, loss of coastal habitats would more than double the shoreline length at high exposure in Kenya and treble in Tanzania; with the former overtaking Mozambique as the second most exposed country, and Tanzania would have the largest population at higher exposure (2.2 million). Coral reefs alone protect 2.5 million people from higher exposure to coastal hazards, mostly in large urban centres.

Under current conditions, 17 (out of 86) coastal districts are considered ‘areas of priority concern’, and four of these are critically exposed (Zavala, Inharrime, Manhiça and Mandlakaze, all in south Mozambique). In these areas, any coastal development would increase the number of people and investment at higher levels of exposure. If coastal habitats are lost, the number of ‘priority concern’ districts would increase to 24. Priority concern districts that show a considerable increase in the population at high exposure if habitats are lost (e.g. Pemba and Mossuril in Mozambique) must prioritise habitat conservation and ecosystem-based management to promote social and environmental resilience. The integrated assessment with regional coverage presented here produced layers of information that can be interrogated to inform risk reduction strategies and decision-making, including allocation of international aid. Applying this knowledge at all administrative levels is timely and urgent in East Africa.

## Supplementary Information

ESM 1 (PDF 924 kb)

ESM 2 (PDF 327 kb)

ESM 3 (PDF 893 kb)

## References

[CR1] Abuodha, P.A. 2009. The African science-base for coastal adaptation: A continental approach. A report to the African Union Commission AUC at the UN Climate Change Conference in Copenhagen (7- 18 Dec 2009). UNESCO-IOC, Available from http://hdl.handle.net/1834/9427. Accessed 11 Feb 2021.

[CR2] Aksha SK, Juran L, Resler LM, Zhang Y (2019). An analysis of social vulnerability to natural hazards in Nepal using a modified social vulnerability index. International Journal of Disaster Risk Science..

[CR3] Arkema KK, Guannel G, Verutes G, Wood SA, Guerry A, Ruckelshaus M, Kareiva P, Lacayo M, Silver JM (2013). Coastal habitats shield people and property from sea-level rise and storms. Nature Climate Change.

[CR4] Arkema KK, Griffin R, Maldonado S, Silver J, Suckale J, Guerry AD (2017). Linking social, ecological, and physical science to advance natural and nature-based protection for coastal communities. Annals of the New York Academy of Sciences.

[CR5] Ashraful Islam M, Mitra D, Dewan A, Akhter SH (2016). Coastal multi-hazard vulnerability assessment along the Ganges deltaic coast of Bangladesh-A geospatial approach. Ocean and Coastal Management.

[CR6] Beck MW, Losada IJ, Menéndez P, Reguero BG, Díaz-Simal P, Fernández F (2018). The global flood protection savings provided by coral reefs. Nature Communications.

[CR7] Becker A, Inoue S, Fischer M, Schwegler B (2012). Climate change impacts on international seaports: Knowledge, perceptions, and planning efforts among port administrators. Climatic Change.

[CR8] Bevacqua A, Yu D, Zhang Y (2018). Coastal vulnerability: Evolving concepts in understanding vulnerable people and places. Environmental Science and Policy.

[CR9] Boruff BJ, Emrich C, Cutter SL (2005). Erosion hazard vulnerability of US coastal counties. Journal of Coastal Research.

[CR10] Brown, S., A.S. Kebede, and R.J. Nicholls. 2009. *Sea level rise and impacts in Africa, 2000 to 2100*. Oxford: Unpublished Report to Stockholm Environment Institute.

[CR11] Burke, L., K. Reytar, M. Spalding, and A. Perry. 2011. *Reefs at risk revisited. Local threats data. Local Threats: Present (KML files)*, World Resources Institute available from https://wriorg.s3.amazonaws.com/s3fs-public/reefs_at_risk_revisited_present.kmz. Accessed 20 Jul 2020.

[CR12] Burke M, Hsiang SM, Miguel E (2015). Global non-linear effect of temperature on economic production. Nature.

[CR13] Cabral P, Augusto G, Akande A, Costa A, Amade N, Niquisse S, Atumane A, Cuna A, Kazemi K, Mlucasse R, Santha R (2017). Assessing Mozambique’s exposure to coastal climate hazards and erosion. International Journal of Disaster Risk Reduction.

[CR14] CES (Consulting Engineers Salzgitter). 2020. *Upscaling nature-based flood protection in Mozambique’s cities, knowledge note*. Washington: World Bank available from http://documents1.worldbank.org/curated/en/401611585291379085/pdf/Upscaling-Nature-Based-Flood-Protection-in-Mozambique-s-Cities-Knowledge-Note.pdf, accessed 20 Jul 2020.

[CR15] Charrua AB, Bandeira SO, Catarino S, Cabral P, Romeiras MM (2020). Assessment of the vulnerability of coastal mangrove ecosystems in Mozambique. Ocean and Coastal Management.

[CR16] Cohen-Shacham, E., G. Walters, C. Janzen, and S. Maginnis, eds. 2016. *Nature-based solutions to address global societal challenges*. Gland, Switzerland: IUCN, 97pp. 10.2305/IUCN.CH.2016.13.en.

[CR17] Cooper JAG, McLaughlin S (1998). Contemporary multidisciplinary approaches to coastal classification and environmental risk analysis. Journal of Coastal Research.

[CR18] Cutter SL, Finch C (2008). Temporal and spatial changes in social vulnerability to natural hazards. Proceedings of the National Academy of Sciences of the United States of America.

[CR19] Cutter SL, Boruff BJ, Shirley WL (2003). Social vulnerability to environmental hazards. Social Science Quarterly.

[CR20] Daly C (2014). A framework for assessing the vulnerability of archaeological sites to climate change: Theory, development, and application. Conservation and Management of Archaeological Sites.

[CR21] Danielsen F, Sørensen MK, Olwig MF, Vaithilingam S, Faizal P, Burgess ND (2005). The Asian tsunami: A protective role for coastal vegetation. Science.

[CR22] Dasgupta, P. 2021. *The economics of biodiversity: The Dasgupta review*. London: HM Treasury.

[CR23] Dasgupta, S., B. Laplante, S. Murray, and D. Wheeler. 2009. *Sea-level rise and storm surges: A comparative analysis of impacts in developing countries*. Washington DC, USA: World Bank.

[CR24] Devi S (2019). Cyclone Idai: 1 month later, devastation persists. Lancet (London, England).

[CR25] Elliff CI, Kikuchi RKP (2015). The ecosystem service approach and its application as a tool for integrated coastal management. Natureza e Conservacao.

[CR26] Faivre N, Sgobbi A, Happaerts S, Raynal J, Schmidt L (2018). Translating the Sendai Framework into action: The EU approach to ecosystem-based disaster risk reduction. International Journal of Disaster Risk Reduction.

[CR27] Fatorić S, Seekamp E (2017). Are cultural heritage and resources threatened by climate change? A systematic literature review. Climatic Change.

[CR28] Fernandino G, Elliff CI, Silva IR (2018). Ecosystem-based management of coastal zones in face of climate change impacts: Challenges and inequalities. Journal of Environmental Management.

[CR29] Fitchett JM, Grab SW (2014). A 66-year tropical cyclone record for south-east Africa: Temporal trends in a global context. International Journal of Climatology.

[CR30] Forino G, MacKee J, von Meding J (2016). A proposed assessment index for climate change-related risk for cultural heritage protection in Newcastle (Australia). International Journal of Disaster Risk Reduction.

[CR31] Francis J, Nilsson A, Waruinge D (2002). Marine protected areas in the Eastern African Region: How successful are they?. Ambio.

[CR32] Gallopín, G.C. 1997. *Indicators and their use: Information for decision-making. Part One-Introduction*, In: Moldan, B. and S. Bilharz (Eds.), Sustainability Indicators. A Report on the Project on Indicators of Sustainable Development, SCOPE 58. Wiley: Chichester, pp.13–27.

[CR33] Ghermandi A, Obura D, Knudsen C, Nunes PALD (2019). Marine ecosystem services in the Northern Mozambique Channel: A geospatial and socio-economic analysis for policy support. Ecosystem Services.

[CR34] Gornitz, V. 1990. Vulnerability of the East Coast, U.S.A. to future sea level rise. *Journal of Coastal Research, *SI 9: 201–237.

[CR35] Gornitz, V., T.W. White, and R.M. Cushman. 1991. *Vulnerability of the US to future sea level rise*, Proceedings of the 7th Symposium on Coastal and Ocean Management: 2354–2368. Available from https://www.osti.gov/servlets/purl/5875484. Accessed 29 Jan 2021.

[CR36] Hahn MB, Riederer AM, Foster SO (2009). The livelihood vulnerability index: A pragmatic approach to assessing risks from climate variability and change-A case study in Mozambique. Global Environmental Change.

[CR37] Hallegatte, S., A. Vogt-Schilb, M. Bangalore, and J. Rozenberg. 2017. *Unbreakable: Building the resilience of the poor in the face of natural disasters. Climate Change and Development*. Washington: World Bank, 187p. Available from: https://openknowledge.worldbank.org/handle/10986/25335. Accessed 11 Feb 2021.

[CR38] Hallegatte, S., J. Rentschler, E. Walsh, and B. James. 2018. *Building back better: Achieving resilience through stronger, faster, and more inclusive post-disaster reconstruction*. Washington: World Bank, 40p. Available from: https://reliefweb.int/sites/reliefweb.int/files/resources/127215.pdf. Accessed 11 Feb 2020.

[CR39] Hammar-Klose, E.S., and E.R. Thieler. 2001. *Coastal vulnerability to sea-level rise, a preliminary database for the U.S. Atlantic, Pacific, and Gulf of Mexico coast*. US Geological Survey Digital Data Series 68. Woods Hole: USGS. 10.3133/ds68.

[CR40] Hinkel J, Brown S, Exner L, Nicholls RJ, Vafeidis AT, Kebede AS (2012). Sea-level rise impacts on Africa and the effects of mitigation and adaptation: An application of DIVA. Regional Environmental Change.

[CR41] Hopper T, Meixler MS (2016). Modeling coastal vulnerability through space and time. PLoS One.

[CR42] Hummell BML, Cutter SL, Emrich CT (2016). Social vulnerability to natural hazards in Brazil. International Journal of Disaster Risk Science.

[CR43] Instituto Nacional de Estadística de Mozambique (INE). 2013. Estatísticas do Distrito. Available from: http://www.ine.gov.mz/estatisticas/estatisticas-territorias-distritais. last access 8 July 2019.

[CR44] Jackson CA, Schmutz P, Harwell MC, Littles CJ (2020). The ecosystem service of property protection and exposure to environmental stressors in the Gulf of Mexico. Ocean and Coastal Management.

[CR45] Kairo JG, Dahdouh-Guebas F, Bosire J, Koedam N (2001). Restoration and management of mangrove systems—a lesson for and from the East African region. South African Journal of Botany.

[CR46] Kebede AS, Nicholls RJ (2012). Exposure and vulnerability to climate extremes: Population and asset exposure to coastal flooding in Dar es Salaam, Tanzania. Regional Environmental Change.

[CR47] Kenya National Bureau of Statistics (KNBS). 2015. County Statistical Abstract. Available from https://www.knbs.or.ke/?page_id=3142 last access July 2019.

[CR48] Kenya National Bureau of Statistics (KNBS) and Society for International Development-East Africa (SID). 2013. Exploring Kenya’s Inequality. Available from https://www.knbs.or.ke/?page_id=3142 last access July 2019.

[CR49] Klinman MG, Reason CJC (2008). On the peculiar storm track of TC Favio during the 2006-2007 Southwest Indian Ocean tropical cyclone season and relationships to ENSO. Meteorology and Atmospheric Physics.

[CR50] Liquete C, Zulian G, Delgado I, Stips A, Maes J (2013). Assessment of coastal protection as an ecosystem service in Europe. Ecological Indicators.

[CR51] Losada, I.J., M. Beck, P. Menendez, A. Espejo, S. Torres, P. Diaz-Simal, F. Fernandez, S. Abad, N. Ripoll, J. García, S. Narayan, D. Trespalacios, and A. Quiroz. 2017. *Valuing protective services of mangroves in the Philippines. Technical report*. Washington: World Bank, 87p. Available from: https://openknowledge.worldbank.org/handle/10986/27666. Accessed 11 Feb 2021.

[CR52] Luijendijk A, Hagenaars G, Ranasinghe R, Baart F, Donchyts G, Aarninkhof S (2018). The state of the world’s beaches. Scientific Reports.

[CR53] Maanan M, Maanan M, Rueff H, Adouk N, Zourarah B, Rhinane H (2018). Assess the human and environmental vulnerability for coastal hazard by using a multi-criteria decision analysis. Human and Ecological Risk Assessment.

[CR54] Mabula, M.J., M.M. Mangora, and C.A. Muhando. 2017. Peri-urban mangroves of Dar es Salaam - Tanzania are highly vulnerable to anthropogenic pressures. *Advances in Ecological and Environmental Research*: 141–172.

[CR55] Macamo, C., S. Bandeira, S. Muando, D. Abreu, and H. Mabilana. 2016. Mangroves of Mozambique. In *Mangroves of the Western Indian Ocean: Status and Management*, ed. Bosire et al., 51–73. Zanzibar Town, Tanzania: WIOMSA.

[CR56] Mahmood R, Ahmed N, Zhang L, Li G (2020). Coastal vulnerability assessment of Meghna estuary of Bangladesh using integrated geospatial techniques. International Journal of Disaster Risk Reduction.

[CR57] Martins CDL, Arantes N, Faveri C, Batista MB, Oliveira EC, Pagliosa PR, Fonseca AL, Nunes JMC, Chow F, Pereira SB, Horta PA (2012). The impact of coastal urbanization on the structure of phytobenthic communities in southern Brazil. Marine Pollution Bulletin.

[CR58] Massuanganhe EA, Macamo C, Westerberg LO, Bandeira S, Mavume A, Ribeiro E (2015). Deltaic coasts under climate-relate catastrophic events - Insights from the Save River delta, Mozambique. Ocean and Coastal Management.

[CR59] Mavromatidi A, Briche E, Claeys C (2018). Mapping and analyzing socio-environmental vulnerability to coastal hazards induced by climate change: An application to coastal Mediterranean cities in France. Cities.

[CR60] Mavume AF, Rydberg L, Rouault M, Lutjeharms JRE (2009). Climatology and landfall of tropical cyclones in the south-west Indian Ocean. Western Indian Ocean Journal of Marine Science.

[CR61] Mazumdar J, Paul SK (2016). Socioeconomic and infrastructural vulnerability indices for cyclones in the eastern coastal states of India. Natural Hazards.

[CR62] Mbonile MJ, Kivelia J (2008). Population, environment and development in Kinondoni District, Dar es Salaam. Geographical Journal.

[CR63] McIvor, A., I. Möller, T. Spencer, and M.D. Spalding. 2012. Reduction of wind and swell waves by mangroves. Natural Coastal Protection Series: Report 1. Cambridge Coastal Research Unit Working Paper 40, 27 p. Available from https://www.wetlands.org/publications/reduction-of-wind-and-swell-waves-by-mangroves/. Accessed 29 Jun 2020.

[CR64] Mclaughlin S, Cooper JAG (2010). A multi-scale coastal vulnerability index: A tool for coastal managers?. Environmental Hazards.

[CR65] Mujabar, P.S., and N. Chandrasekar. 2013. Coastal erosion hazard and vulnerability assessment for southern coastal Tamil Nadu of India by using remote sensing and GIS. *Natural Hazards* 69 (3): 1295–1314. 10.1007/s11069-011-9962-x.

[CR66] Mutombo K, Ölçer A (2017). Towards port infrastructure adaptation: A global port climate risk analysis. WMU Journal of Maritime Affairs.

[CR67] National Bureau of Statistics, Tanzania (NBS). 2016. Basic Demographic and Socio-Economic Profile. Population and Housing Census 2012. Available from https://www.nbs.go.tz/index.php/en/regional-profiles ,last access August 2019.

[CR68] Neumann B, Vafeidis AT, Zimmermann J, Nicholls RJ (2015). Future coastal population growth and exposure to sea-level rise and coastal flooding - A global assessment. PLoS One.

[CR69] Nguyen TTX, Bonetti J, Rogers K, Woodroffe CD (2016). Indicator-based assessment of climate-change impacts on coasts: A review of concepts, methodological approaches and vulnerability indices. Ocean and Coastal Management.

[CR70] Nguyen KA, Liou YA, Terry JP (2019). Vulnerability of Vietnam to typhoons: A spatial assessment based on hazards, exposure and adaptive capacity. Science of the Total Environment.

[CR71] Niang, I., O.C. Ruppel, M.A. Abdrabo, A. Essel, C. Lennard, J. Padgham, and P. Urquhart. 2014. Chapter 22 - Africa. In: *Climate Change 2014: Impacts, adaptation and vulnerability: Part B: Regional Aspects. Contributions of the Working Group II to the Fifth Assessment Report of the Intergovernmental Panel on Climate Change*. Cambridge: Cambridge University Press, pp. 1199-1266. Available from: 10.1017/CBO9781107415386.002. Accessed 20 Jun 2020.

[CR72] Nursey-Bray M, Blackwell B, Brooks B, Campbell ML, Goldsworthy L, Pateman H, Rodrigues I, Roome M, Wright JT, Francis J, Hewitt CL (2013). Vulnerabilities and adaptation of ports to climate change. Journal of Environmental Planning and Management.

[CR73] Obura D (2012). The diversity and biogeography of Western Indian Ocean reef-building corals. PLoS One.

[CR74] Obura DO, Wells S, Church J, Horrill C (2002). Monitoring of fish and fish catches by local fishermen in Kenya and Tanzania. Marine and Freshwater Research.

[CR75] OECD 2019. Development Aid at a Glance, Statistics by Region, 2. Africa. Available from https://www.oecd.org/dac/financing-sustainable-development/development-finance-data/Africa-Development-Aid-at-a-Glance-2019.pdf, last accessed 10 Jul 2020.

[CR76] Ogie RI, Pradhan B (2019). Natural hazards and social vulnerability of place: The strength-based approach applied to Wollongong, Australia. International Journal of Disaster Risk Science.

[CR77] Onat Y, Marchant M, Francis OP, Kim K (2018). Coastal exposure of the Hawaiian islands using GIS-based index modeling. Ocean and Coastal Management.

[CR78] Oulahen G, McBean G, Shrubsole D, Chang SE (2019). Production of risk: Multiple interacting exposures and unequal vulnerability in coastal communities. Regional Environmental Change.

[CR79] Phillips H (2015). The capacity to adapt to climate change at heritage sites-The development of a conceptual framework. Environmental Science and Policy.

[CR80] Rabby YW, Hossain MB, Hasan MU (2019). Social vulnerability in the coastal region of Bangladesh: An investigation of social vulnerability index and scalar change effects. International Journal of Disaster Risk Reduction.

[CR81] Rakotobe, T. 2012. *Climate change in the Western Indian Ocean: A situation assessment and policy considerations*. 4245 North Fairfax Drive, Suite 100, Arlington, VA,22203: Africa Biodiversity Collaborative Group Report October 2012, 118pp.

[CR82] Ran J, MacGillivray BH, Gong Y, Hales TC (2020). The application of frameworks for measuring social vulnerability and resilience to geophysical hazards within developing countries: A systematic review and narrative synthesis. Science of the Total Environment.

[CR83] Rao NS, Ghermandi A, Portela R, Wang X (2015). Global values of coastal ecosystem services: A spatial economic analysis of shoreline protection values. Ecosystem Services.

[CR84] Reeder-Myers LA (2015). Cultural heritage at risk in the twenty-first century: A vulnerability assessment of coastal archaeological sites in the United States. Journal of Island and Coastal Archaeology.

[CR85] Renaud, F.G., K. Sudmeier-Rieux, M. Estrella, and U. Nehren. 2016. *Ecosystem-based disaster risk reduction and adaptation in practice, Advances in Natural and Technological Hazards Research 42*. New York: Springer.

[CR86] Romão X, Paupério E, Pereira N (2016). A framework for the simplified risk analysis of cultural heritage assets. Journal of Cultural Heritage.

[CR87] Ruckelshaus MH, Guannel G, Arkema K, Verutes G, Griffin R, Guerry A, Silver J, Faries J, Brenner J, Rosenthal A (2016). Evaluating the benefits of green infrastructure for coastal areas: Location, location, location. Coastal Management.

[CR88] Sabai D (2017). Disambiguating praxis from practice in natural resource management: A practical space for enhancing experiential learning in the eastern coast of Tanzania. Transylvanian Review of Systematical and Ecological Research.

[CR89] Sajjad M, Chan JCL (2019). Risk assessment for the sustainability of coastal communities: A preliminary study. Science of the Total Environment.

[CR90] Sajjad M, Chan JCL, Kanwal S (2020). Integrating spatial statistics tools for coastal risk management: A case-study of typhoon risk in mainland China. Ocean and Coastal Management.

[CR91] Samoilys, M., M. Pabari, T. Andrew, M. Waweru, J. Church, A. Momayi, B. Mibei, M. Monjane, A. Shah, M. Menomussanga, and D. Mutta. 2015. *Resilience of coastal systems and their human partners: Ecological and social profile of coastal systems in Kenya, Mozambique and Tanzania*. Nairobi, Kenya: IUCN ESARO, WIOMSA, CORDIO and the UNEP Nairobi Convention.

[CR92] Schneiderbauer, S., and D. Ehrlich. 2004. *Risk, hazard and people’s vulnerability to natural hazards. A review of definitions, concepts and data. Report No: EUR21410*. Join Research Centre, European Commission, 40 p. Available from: https://www.researchgate.net/publication/268149143_Risk_Hazard_and_People's_Vulnerability_to_Natural_Hazards_a_Review_of_Definitions_Concepts_and_Data. Accessed 8 Feb 2021.

[CR93] Serafim MB, Siegle E, Corsi AC, Bonetti J (2019). Coastal vulnerability to wave impacts using a multi-criteria index: Santa Catarina (Brazil). Journal of Environmental Management.

[CR94] Sharp, R., H. T. Tallis, T. Ricketts, A. D. Guerry, S. A. Wood, R. Chaplin-Kramer, E. Nelson, D. Ennaanay, S. Wolny, N. Olwero, K. Vigerstol, D. Pennington, G. Mendoza, J. Aukema, J. Foster, J. Forrest, D. Cameron, K. Arkema, E. Lonsdorf, C. Kennedy, G. Verutes, C.K. Kim, G. Guannel, M. Papenfus, J. Toft, M. Marsik, J. Bernhardt, R. Griffin, K. Glowinski, N. Chaumont, A. Perelman, M. Lacayo, L. Mandle, P. Hamel, A.L. Vogl, L. Rogers, W. Bierbower, D. Denu, and J. Douglass. 2020. InVEST 3.8. User Guide. Available from: https://invest-userguide.readthedocs.io/en/3.8.2/coastal_vulnerability.html. Accessed 20 May 2020.

[CR95] Silva R, Lithgow D, Esteves LS, Martínez ML, Moreno-Casasola P, Martell R, Pereira P, Mendoza E, Campos-Cascaredo A, Winckler Grez P, Osorio AF, Osorio-Cano JD, Rivillas GD (2017). Coastal risk mitigation by green infrastructure in Latin America. Maritime Engineering.

[CR96] Silver JM, Arkema KK, Griffin RM, Lashley B, Lemay M, Maldonado S, Moultrie SH, Ruckelshaus M, Schill S, Thomas A, Wyatt K, Verutes G (2019). Advancing coastal risk reduction science and implementation by accounting for climate, ecosystems, and people. Frontiers in Marine Science.

[CR97] Spalding, M., M. Kainuma, and L. Collins. 2010. *World Atlas of Mangroves (version 3.0). A collaborative project of ITTO, ISME, FAO, UNEP-WCMC, UNESCO-MAB, UNU-INWEH and TNC*. London: Earthscan, 319p. 10.34892/w2ew-m835.

[CR98] Spalding MD, Ruffo S, Lacambra C, Meliane I, Hale LZ, Shepard CC, Beck MW (2014). The role of ecosystems in coastal protection: Adapting to climate change and coastal hazards. Ocean and Coastal Management.

[CR99] Stewart, M., and S. Fairfull. 2008. *Mangroves. Primefact 746,* NSW Department of Primary Industries, State of New South Wales, Australia,16p. Available from: http://www.dpi.nsw.gov.au/__data/assets/pdf_file/0020/236234/mangroves.pdf. Accessed 16 Sep 2020.

[CR100] Tapsell SM, Penning-Rowsell EC, Tunstall SM, Wilson TL (2002). Vulnerability to flooding: Health and social dimensions. Philosophical transactions Series A, Mathematical, physical, and engineering sciences.

[CR101] UN General Assembly (UNGA). 2016. *Report of the open-ended intergovernmental expert working group on indicators and terminology relating to disaster risk reduction*, 41. New York, NY, USA: United Nations General Assembly.

[CR102] UNDP. 2020. Human Development Index Ranking. Available from http://hdr.undp.org/en/content/2019-human-development-index-ranking, accessed 21 Jul 2020.

[CR103] UNEP-Nairobi Convention. 2009. *Transboundary diagnostic analysis of land-based sources and activities affecting the Western Indian Ocean coastal and marine environment*. Nairobi: UNEP, 378p. Available from: https://www.unep.org/resources/report/transboundary-diagnostic-analysis-land-based-sources-and-activities-western-indian. Accessed 30 Jul 2020.

[CR104] UNEP-Nairobi Convention and WIOMSA. 2015. *Regional state of the coast report: Western Indian Ocean*. Nairobi, Kenya: UNEP and WIOMSA 546pp. Available from http://hdl.handle.net/20.500.11822/9668. Accessed 30 Jul 2020.

[CR105] UNEP-WCMC, Short FT. 2017. *Global distribution of seagrasses (version 6.0). Sixth update to the data layer used in Green and Short (2003)*. Cambridge: UN Environment World Conservation Monitoring Centre. 10.34892/x6r3-d211. Accessed 20 Jan 2020.

[CR106] UNEP-WCMC, World Fish Centre, WRI, and TNC. 2018. *Global distribution of coral reefs, compiled from multiple sources including the Millennium Coral Reef Mapping Project. Version 4.0, updated by UNEP-WCMC. Includes contributions from IMaRSUSF and IRD (2005), IMaRS-USF (2005) and Spalding et al. (2001)*. Cambridge: UNEP World Conservation Monitoring Centre. 10.34892/t2wk-5t34. Accessed 25 Jan 2020.

[CR107] UNISDR. 2015. *Making development sustainable: the future of disaster risk management. Global assessment report on disaster risk reduction*. Geneva, Switzerland: United Nations Office for Disaster Risk Reduction (UNISDR).

[CR108] UNISDR. 2016. DesInventar. Disaster Information System, United Nations Office for Disaster Risk Reduction. http://www.desinventar.net/ last access May 2020

[CR109] United Nations, Department of Economic and Social Affairs, Population Division. 2019. World Population Prospects 2019: Highlights. ST/ESA/SER.A/423. Available from https://population.un.org/wpp/Publications/Files/WPP2019_Highlights.pdf last accessed 19 Jul 2020.

[CR110] Vincent, K. 2004. *Creating an index of social vulnerability to climate change for Africa*. Tyndall Centre Working paper 56, Norwich: University of East Anglia, 51p.

[CR111] Vojinovic Z, Hammond M, Golub D, Hirunsalee S, Weesakul S, Meesuk V, Medina N, Sanchez A, Kumara S, Abbott M (2016). Holistic approach to flood risk assessment in areas with cultural heritage: A practical application in Ayutthaya, Thailand. Natural Hazards.

[CR112] Wagner, G.M. 2004. Coral reefs and their management in Tanzania. *Western Indian Ocean Journal of Marine Science* 3: 227–243. 10.4314/wiojms.v3i2.28464.

[CR113] Wagner GM (2005). Participatory monitoring of changes in coastal and marine biodiversity. Indian Journal of Marine Sciences.

[CR114] Wagner, G. 2008. The Dar es Salaam seascape: A case study of an environmental management ‘hotspot’. *Western Indian Ocean Journal of Marine Science* 6 (1). 10.4314/wiojms.v6i1.48229.

[CR115] Welle, T., M. W. Beck, and J. Birkmann. 2014. The coasts at risk index. In: M.W. Beck (ed.), An assessment of coastal risks and the role of environmental solutions, United Nations University-EHS, The Nature Conservancy and the University of Rhode Island-CRC, 5-24pp. Available from: https://www.conservationgateway.org/ConservationPractices/Marine/crr/library/Documents/Coasts_at_Risk_2014.pdf. Accessed 2 Feb 2021.

[CR116] Wells S, Burgess N, Ngusaru A (2007). Towards the 2012 marine protected area targets in Eastern Africa. Ocean and Coastal Management.

[CR117] Zacarias DA (2019). Understanding community vulnerability to climate change and variability at a coastal municipality in southern Mozambique. International Journal of Climate Change Strategies and Management.

[CR118] Zhang Y, Ruckelshaus M, Arkema KK, Han B, Lu F, Zheng H, Ouyang Z (2020). Synthetic vulnerability assessment to inform climate-change adaptation along an urbanized coast of Shenzhen, China. Journal of Environmental Management.

