# Supplementary material for: Integrated Assessment of Coastal Exposure and Social Vulnerability to Coastal Hazards in East Africa
Source: Estuaries Coast. 2021 May 13;44(8):2056–72. doi: 10.1007/s12237-021-00930-5 (PMC8118621; doi:10.1007/s12237-021-00930-5)
Supplement: Supplementary file 1 — (PDF 924 kb) [file 12237_2021_930_MOESM1_ESM.pdf]

Electronic Supplementary Material 1. Data manipulation

Integrated assessment of coastal exposure and social vulnerability to coastal hazards in East Africa

Estuaries and Coasts

Dr Caridad Ballesteros, cballesteros@bournemouth.ac.uk

Dr Luciana S. Esteves, lesteves@bournemouth.ac.uk (corresponding author)

Department of Life & Environmental Sciences

Faculty of Science & Technology

Bournemouth University

Fern Barrow, Poole, BH12 5BB, UK

Improving the accuracy of the shoreline

One of the data inputs required by the InVEST coastal vulnerability model is the "Landmass". The Landmass is a polygon representing the contour of the land, which the model uses to identify the shoreline (i.e. the boundary between the sea and land). The model provides a global landmass (Wessel and Smith 1996) as the default dataset but users are encouraged to use files that more accurately represent the area of interest. To this end, we have used open data available from GADM (Global Administrative Areas 2019), as it was found to more accurately reflect the land-sea boundary in East Africa than the default dataset available within InVEST (Fig. S1). GADM provides high-resolution spatial data of administrative boundaries for all countries and their subdivisions. However, the vector boundaries they provide show a pixelated serrated shape, which can be smoothed in GIS to more closely represent the shoreline. We have used the "Smoothing polygon" tool in ArcMap 10.1 selecting the "Smoothing Algorithm PAEK" and a smoothing tolerance of 130m. Using this procedure, the undesirable serrated shape was eliminated (Fig. S1); and this improved 'landmass' was then used as the model input.

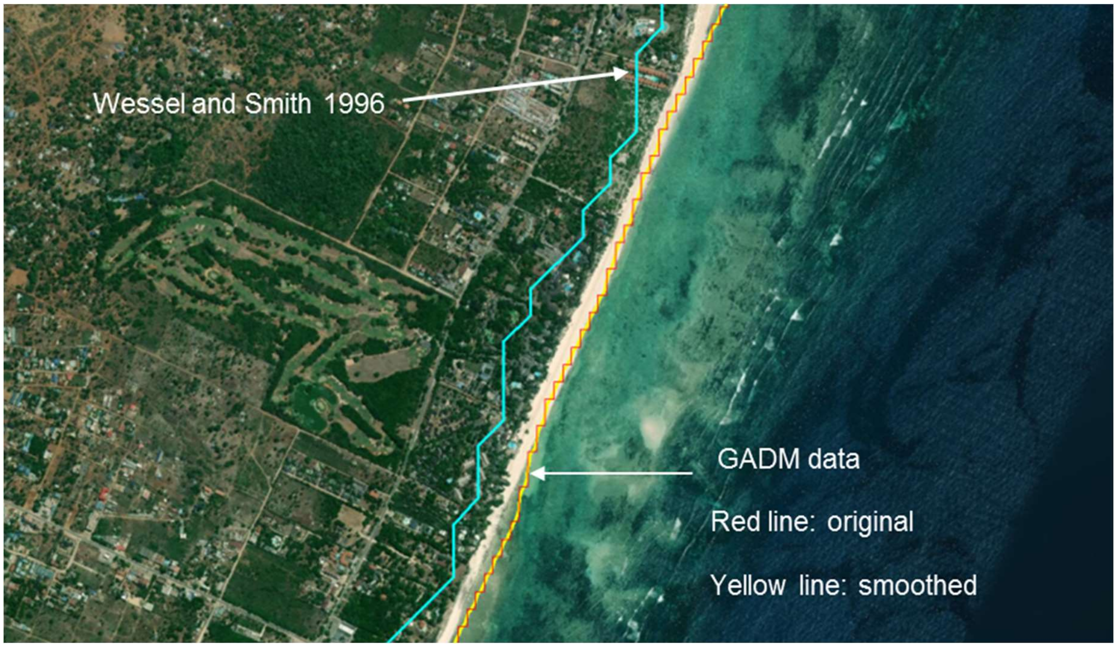

Fig. S1. 'Landmass' boundaries of the default dataset (Wessel and Smith 1996) embedded in the InVEST model (in blue) and the GADM data in its original form (in red) and after 'smoothing' (in yellow). Source: Esri, DigitalGlobe, GeoEye, Earthstar Geographics, CNES/Airbus DS, USDA, USGS, AeroGRID, IGN, and the GIS User Community.

Shoreline change rates

Shoreline change rate data obtained from Luijendijk et al. (2018) did not completely fit the coastal exposure data, as shoreline change data were distributed every 500m along the coast and around 1km inland, whereas the coastal exposure data were distributed every 1km along the coastline. In order to include the shoreline change rate data within the coastal exposure, we used a spatial analysis in which the closest shoreline rate data point was joined to each of the coastal exposure points. Where the join between the shoreline change rate and coastal exposure point showed a distance greater than 2km, it was considered that no erosion data is

available for that coastal exposure point and a value of 5 was assigned to the erosion component as the worst case scenario.

Habitats users-defined radius of protection

Table S1. Radius of protection for coastal habitats

Coastal habitat	Radius of protection
Coral Reefs	2000 m
Mangroves	2000 m
Seagrasses	500 m

Social Vulnerability Index

The social vulnerability index (SVI) combines indicators that reflect the degree to which a community is prone to the impacts of natural hazards. The selection of indicators was informed by a literature review to identify the most commonly used in previous studies focusing on social vulnerability to natural hazards in developing countries (e.g. Mazumdar and Paul 2018; Aksha et al. 2019; Zacarias 2019). The indicators most commonly used that were available for the study area and collected/reported in comparable ways were then selected to comprise the SVI. Madagascar was excluded from this assessment as the latest census data available dates from 1993 and was deemed too outdated to be comparable with the other three countries (Kenya, Tanzania and Mozambique). Eight indicators were used to produce the SVI, with one of them (the proportion of houses made with natural materials) aggregating three sub-indicators (natural materials used wall, roof and floor), by taking the average of their values (Table S2). The data were collated from the latest available census and the official statistics provided by each country at district level (or higher levels if unavailable). Where needed to enable comparison across districts, each indicator was represented as a percentage of the respective total population or total number of houses.

Vulnerable age is one of the most used demographic drivers in social vulnerability assessment (Rufat et al. 2015). Vulnerable age groups are defined, in the context of natural hazards, as people younger than five and older than 65 years (Sorg et al. 2018). Extreme age may have constraints in mobility, increasing the liability of care at the time of the hazard (e.g. Cutter et al. 2003; Mazumdar and Paul 2016; Sorg et al. 2018; Aksha et al. 2019). Non- masonry houses have a significant impact on vulnerability to natural hazards as they can be easily destroyed (Mazumdar and Paul 2016; Aksha et al. 2019). Hummel et al. 2016, p.116 also included indicators equivalent to households without access to improved drinking water or toilet facilities and explained that "after a natural disaster, these households could require more time recovering, and would be at greater risk of soil and water contamination." Another commonly used demographic indicator is population density. A large population in the same area suggests not only that more people would be affected by a disaster, but also that they would have more difficulty in an eventual evacuation or rescue situation, making them more vulnerable to the natural hazard (Hummell et al. 2016). Rapid population growth, as seen in many developing countries, also puts pressure on the lack of available quality housing, quality of life, basic services etc. (e.g. Cutter et al. 2003; Hummell et al. 2016; Mazumdar and Paul 2016; Sorg et al. 2018). Illiteracy impacts on the ability of the population to understand, and act upon, warning systems and access recovery information. (e.g. Cutter et. al 2003; Mazumdar and Paul 2016)

Table S2 provides further information about the sources of data and the composition of each indicator.

Table S2. Indicators used in the social vulnerability index, their sub-indicators (where relevant) and data sources.

Indicators	Mozambique	Tanzania	Kenya
**Source of data**	Estatísticas Distritais 2013. (Data based on the 'Recenseametno geral da população e habitação 2007)	Basic Demographic and Socio-economic profile 2016. 2012 Population and Housing Census (PHC) for the United Republic of Tanzania	Exploring Kenya's Inequality 2013 Kenya National Bureau of Statistics (KNBS) and Society for International Development (SID) - Based on 2009 Kenya housing and population census.
**1. % houses made with natural materials**	Average of sub-indicators a, b and c<br>*Materials included in each sub-indicator, as written in the original source (in Portuguese for Mozambique)*		
a. % houses with "Wall" made with natural materials	- Bloco de adobe<br>- Caniço/paus/bambú/palmeira<br>- Paus maticados<br>- Lata/cartão/papel/saco/casca<br>- Outros	- Timber<br>- Timber and Iron Sheets<br>- Poles and Mud<br>- Grass<br>- Canvass	- Mud/Wood<br>- Corrugated Iron Sheets<br>- Grass/ Reeds<br>- Wood only<br>- Tin<br>- Other
b. % houses with "Roof" made with natural materials	- Capim/colmo/palmeira<br>- Chapa de lusalite<br>- Chapa de zinco<br>- Outros	- Grass/ Leaves<br>- Mud and Leaves<br>- Plastics /Box Paper Canvas<br>- Iron Sheets	- Grass<br>- Makuti<br>- Tin<br>- Mud/Dung<br>- Iron sheets<br>- Other
c. % houses with "Floor" made with natural materials	- Adobe<br>- Sem nada<br>- Outros	- Wood Planks<br>- Palm/ Bamboo Planks<br>- Earth/ Sand<br>- Animal Dung	- Wood<br>- Earth<br>- Other
**2. % houses without improved drinking water sources**	- Poço sem bomba (céu aberto)<br>- Rio / Lago / Lagoa	- Unprotected dug well<br>- Unprotected Spring<br>- Rain water collection<br>- Bottled water<br>- Cart with small tank/drum<br>- Tanker truck<br>- Surface water (river dam lake etc.)	- Unprotected Spring<br>- Unprotected Well<br>- Pond<br>- Dam<br>- Lake<br>- Stream/River<br>- Jabia<br>- Water vendor<br>- Other
**3. % houses without improved toilet facility**	- Latrina Tradicional Não Melhorada<br>- Sem Latrina	- Flush/Pour water to Somewhere Else<br>- Pit Latrine without Washable/ Soil Slab<br>- Pit Latrine without Slab/Open Pit<br>- Bucket<br>- No Facility/ bush/ field/ beach	- Pit Latrine uncovered<br>- Bucket<br>- Bush<br>- Other
**4. % Illiterate population**	% Illiterate population above 15 years old (source: "Indicadores Socio-Demográficos Distritais Recenseamento Geral Da População E Habitação 2007")	100-%Literacy rate above 15 years old	%Illiteracy
**5. Population density**	Population/Area (km^2)	Population/Area (km^2)<br><br>(Area obtained from GADM Administrative divisions - WGS 1984 UTM Zone 37S)	Population/Area (km^2)<br><br>(Area obtained from GADM Administrative divisions - WGS 1984 UTM Zone 37S)
**6. Population growth rate per annum**	Period: 2009-2014	Period: 2002-2012	Period: 2010-2014<br>(data at County level, extracted from "KNBC (2015) County Statistical Abstract -2015")
**7. % young children**	% population aged < 4 years old	% population aged < 4 years old (data at Region level, administrative level 1)	% population aged < 5 years old

8. % elderly	% population aged > 65 years old	% population aged > 65 years old (data at Region level, administrative level 1)	% population aged > 65 years old

The data treatment employed to build the SVI followed approaches used in other social vulnerability indices (e.g. Tapsell et al. 2002). The eight indicators and their percentages were transformed by natural logarithm to minimise the skewness and kurtosis within their distributions. Following this transformation, the values were standardized as Z-Scores and then summed. The sum of the Z-Scores obtained at the district level ranged from -24.72 to 4.73. These were ranked into five classes using a quantile interval (Table S3).

Table S3. SVI ranking classes based on the quartile distribution of the sum of their normalised Z-Scores

SVI Ranking	Sum of the indicators Z-Scores
1 - Very low	< -2.66
2 - Low	-2.66 - 0.49
3 - Moderate	0.49 - 2.03
4 - High	2.03 - 2.74
5 - Very high	>2.74

References:

Aksha, S. K., Juran, L., Resler, L. M., and Zhang, Y. 2019. An Analysis of Social Vulnerability to Natural Hazards in Nepal Using a Modified Social Vulnerability Index. *International Journal of Disaster Risk Science*. 10:103–116. doi:10.1007/s13753-018-0192-7.

Cutter, S.L., Boruff, B.J. and W. L. Shirley. 2003. Social vulnerability to environmental hazards. *Social Science Quarterly* 84: 242–261. doi:10.1111/1540-6237.8402002.

Hummell, B.M.L., Cutter, S.L. and Emrich, C.T. 2016. Social Vulnerability to Natural Hazards in Brazil. *International Journal of Disaster Risk Science* 7:111–122. doi.org/10.1007/s13753-016-0090-9

Global Administrative Areas (GADM). 2019. Database of Global Administrative Areas, http://www.gadm.org.

Instituto Nacional de Estadística de Mozambique (INE). 2013. Estatísticas do Distrito. Available from: http://www.ine.gov.mz/estatisticas/estatisticas-territorias-distritais. Last access 8 July 2019.

Kenya National Bureau of Statistics (KNBS) and Society for International Development-East Africa (SID). 2013. Exploring Kenya's Inequality. Available from https://www.knbs.or.ke/?page_id=3142 last access July 2019

Kenya National Bureau of Statistics (KNBS). 2015. County Statistical Abstract. Available from https://www.knbs.or.ke/?page_id=3142 last access July 2019

Luijendijk, A., G. Hagenaars, R. Ranasinghe, F. Baart, G. Donchyts, and S. Aarninkhof. 2018. The State of the World's Beaches. *Scientific Reports* 1–11. doi:10.1038/s41598-018-24630-6.

Mazumdar, J. and S.K. Paul. 2018. A spatially explicit method for identification of vulnerable hotspots of Odisha, India from potential cyclones. *International Journal of Disaster Risk Reduction* 27:391–405. doi.org/10.1016/j.ijdrr.2017.11.001.

Mazumdar, J., and S. K. Paul. 2016. Socioeconomic and infrastructural vulnerability indices for cyclones in the eastern coastal states of India. *Natural Hazards* 82: 1621–1643. doi:10.1007/s11069-016-2261-9.

National Bureau of Statistics, Tanzania (NBS). 2016. Basic Demographic and Socio-Economic Profile. Population and Housing Census 2012. Available from https://www.nbs.go.tz/index.php/en/regional-profiles Last access August 2019.

Rufat, S., E. Tate, C.G. Burton, and A.S. Maroof. 2015. Social vulnerability to floods: Review of case studies and implications for measurement. *International Journal of Disaster Risk Reduction* 14(Part 4): 470–486.

Sorg, L., Medina, N., Feldmeyer, D., Arlex S., Vojinovic, Z., Birkmann, J., and A. Marchese. 2018. Capturing the multifaceted phenomena of socioeconomic vulnerability. *Natural Hazards* 92: 257–282. doi.org/10.1007/s11069-018-3207-1

Tapsell, S. M., E. C. Penning-Rowsell, S. M. Tunstall, and T. L. Wilson. 2002. Vulnerability to flooding: health and social dimensions. *Philosophical transactions Series A, Mathematical, physical, and engineering sciences* 360: 1511–25. doi:10.1098/rsta.2002.1013.

Zacarias, D. A. 2019. Understanding community vulnerability to climate change and variability at a coastal municipality in southern Mozambique. *International Journal of Climate Change Strategies and Management* 11: 154–176. doi:10.1108/IJCCSM-07-2017-0145.
