# Supplementary material for: Integrated Assessment of Coastal Exposure and Social Vulnerability to Coastal Hazards in East Africa
Source: Estuaries Coast. 2021 May 13;44(8):2056–72. doi: 10.1007/s12237-021-00930-5 (PMC8118621; doi:10.1007/s12237-021-00930-5)
Supplement: Supplementary file 2 — (PDF 327 kb) [file 12237_2021_930_MOESM2_ESM.pdf]

Electronic Supplementary Material 2. Validation data

Integrated assessment of coastal exposure and social vulnerability to coastal hazards in East Africa

Estuaries and Coasts

Dr Caridad Ballesteros, cballesteros@bournemouth.ac.uk

Dr Luciana S. Esteves, lesteves@bournemouth.ac.uk (corresponding author)

Department of Life & Environmental Sciences

Faculty of Science & Technology

Bournemouth University

Fern Barrow, Poole, BH12 5BB, UK

Table S4. Data used in the validation included number of events and number of events causing deaths at district level, % of district shoreline length at higher exposure, classification of exposed and not-exposed districts using thresholds of 20%, 25%, 30%

Country	District	Number of Events causing deaths	Number of Events	>20% coastline at higher exposure (1 = yes)	>25% coastline at higher exposure (1 = yes)	>30% coastline at higher exposure (1 = yes)	>50% coastline at higher exposure (1 = yes)	% coastline at higher exposure (Scenario 1)	SVI	IVCC (Scenario 1)
Kenya	Kilifi	6	20	1	0	0	0	22.10	3	5.74
Kenya	Kwale	1	4	0	0	0	0	7.14	4	5.91
Kenya	Lamu	0	3	0	0	0	0	10.10	3	5.24
Kenya	Mombasa	3	7	0	0	0	0	10.07	1	3.29
Kenya	Tana River	1	20	1	1	1	1	64.65	5	8.05
Madagascar	Ambanja	5	8	0	0	0	0	9.37		
Madagascar	Ambilobe	1	7	1	1	1		43.81		
Madagascar	Amboasary-Sud	1	3	1	1	1	1	81.48		
Madagascar	Ambovombe-Androy	1	3	1	1	1	1	90.91		
Madagascar	Ampanihy	2	5	0	0	0	0	15.55		
Madagascar	Ampasimanolotra	4	8	1	1	1	1	100.00		
Madagascar	Analalava	3	8	0	0	0	0	3.56		
Madagascar	Antalaha	7	8	0	0	0	0	18.52		
Madagascar	Antsalova	0	2	1	1	0	0	29.63		
Madagascar	Antsihihy	2	7	0	0	0	0	0.00		
Madagascar	Antsiranana Rural	1	3	0	0	0	0	5.49		
Madagascar	Beloha	0	2	1	1	1	1	65.71		
Madagascar	Belon-i Tsiribihina	1	6	1	0	0	0	20.34		
Madagascar	Besalampy	3	4	1	1	1	0	40.93		
Madagascar	Betioky-Sud	3	4	0	0	0	0	5.05		
Madagascar	Farafangana	9	11	1	1	1	1	100.00		
Madagascar	Fenoarivo Atsinanana	2	8	1	1	1	1	67.14		
Madagascar	Mahajanga Rural	4	12	0	0	0	0	4.91		

Country	District	Number of Events causing deaths	Number of Events	>20% coastline at higher exposure (1 = yes)	>25% coastline at higher exposure (1 = yes)	>30% coastline at higher exposure (1 = yes)	>50% coastline at higher exposure (1 = yes)	% coastline at higher exposure (Scenario 1)	SVI	IVCC (Scenario 1)
Madagascar	Mahajanga Urban	3	7	0	0	0	0	14.29		
Madagascar	Mahanoro	3	5	1	1	1	1	50.77		
Madagascar	Maintirano	3	4	1	1	0	0	28.22		
Madagascar	Manakara-Sud	7	12	1	1	1	1	60.16		
Madagascar	Mananara	5	10	1	1	0	0	26.09		
Madagascar	Mananjary	6	9	1	1	1	1	77.55		
Madagascar	Manja	1	4	1	1	0	0	28.19		
Madagascar	Maroansetra	5	9	1	1	1	0	38.17		
Madagascar	Mitsinjo	0	4	0	0	0	0	10.35		
Madagascar	Morombe	3	7	0	0	0	0	9.00		
Madagascar	Morondava	4	6	1	0	0	0	21.91		
Madagascar	Nosibe	1	4	0	0	0	0	2.23		
Madagascar	Nosy varika	2	8	1	1	1	1	67.37		
Madagascar	Nosy-Boraha (St. Marie)	1	6	1	1	0	0	28.66		
Madagascar	Port Bergé	3	8	0	0	0	0	8.05		
Madagascar	Sambava	3	7	1	1	1	1	68.60		
Madagascar	Soalala	1	6	0	0	0	0	18.64		
Madagascar	Soanierana-Ivongo	3	8	1	1	1	1	69.37		
Madagascar	Taolagnaro	3	6	1	1	1	0	39.63		
Madagascar	Toamasina Rural	7	15	1	1	1	1	82.81		
Madagascar	Toliary	3	4	0	0	0	0	3.23		
Madagascar	Toliary Urban	3	4	0	0	0	0	4.17		
Madagascar	Tsiombe	0	1	1	1	1	1	88.37		
Madagascar	Vangaindrano	11	13	1	1	1	1	63.68		
Madagascar	Vatomandry	3	8	1	1	1	1	90.54		
Madagascar	Vohimarina (Iharana)	3	4	0	0	0	0	12.11		

Country	District	Number of Events causing deaths	Number of Events	>20% coastline at higher exposure (1 = yes)	>25% coastline at higher exposure (1 = yes)	>30% coastline at higher exposure (1 = yes)	>50% coastline at higher exposure (1 = yes)	% coastline at higher exposure (Scenario 1)	SVI	IVCC (Scenario 1)
Madagascar	Vohipeno	7	11	1	1	1	1	82.50		
Mozambique	Ancuabe	0	6	0	0	0	0	0.00	5	6.85
Mozambique	Angoche	4	19	0	0	0	0	7.31	5	7.22
Mozambique	Bilene - Macia	2	16	1	1	1	0	41.18	3	5.53
Mozambique	Boane	3	10	0	0	0	0	0.00	3	5.82
Mozambique	Buzi	10	25	1	1	0	0	26.59	4	6.55
Mozambique	Cheringoma	1	6	1	1	1	0	38.71	5	7.69
Mozambique	Chinde	3	20	1	1	1	0	37.68	4	6.81
Mozambique	Chiure	0	10	0	0	0	0	0.00	4	5.96
Mozambique	Dondo	15	43	1	1	1	1	63.73	2	5.13
Mozambique	Govuro	5	11	0	0	0	0	18.62	4	6.39
Mozambique	Homoine	0	10	0	0	0	0	14.30	5	7.50
Mozambique	Inharrime	1	6	1	1	1	1	90.74	5	8.18
Mozambique	Inhassoro	1	7	1	1	1	0	35.97	3	5.68
Mozambique	Inhassunge	4	8	0	0	0	0	11.37	4	6.22
Mozambique	Jangamo	1	20	0	0	0	0	14.20	5	7.46
Mozambique	Machanga	4	20	0	0	0	0	9.07	4	6.31
Mozambique	Macomia	0	13	0	0	0	0	4.70	4	6.29
Mozambique	Maganja Da Costa	8	20	1	1	1	1	52.38	4	7.11
Mozambique	Mandlacaze	3	13	1	1	1	1	100.00	4	7.32
Mozambique	Manhiça	4	24	1	1	1	1	100.00	5	8.25
Mozambique	Maputo	2	8	1	1	0	0	27.20	1	3.70
Mozambique	Marracuene	2	2	1	1	1	1	52.89	3	5.97
Mozambique	Marromeu	6	20	1	1	0	0	28.35	4	6.56
Mozambique	Massinga	3	8	1	1	1	1	53.62	3	5.75
Mozambique	Matutuine	0	0	1	1	0	0	26.50	2	4.57

Country	District	Number of Events causing deaths	Number of Events	>20% coastline at higher exposure (1 = yes)	>25% coastline at higher exposure (1 = yes)	>30% coastline at higher exposure (1 = yes)	>50% coastline at higher exposure (1 = yes)	% coastline at higher exposure (Scenario 1)	SVI	IVCC (Scenario 1)
Mozambique	Mecufi	2	9	0	0	0	0	11.76	4	6.38
Mozambique	Memba	1	6	0	0	0	0	0.00	5	6.95
Mozambique	Mocimboa Da Praia	1	11	0	0	0	0	15.71	4	6.42
Mozambique	Mogincual	2	8	0	0	0	0	11.73	5	7.74
Mozambique	Moma	7	20	1	1	1	0	33.33	5	7.18
Mozambique	Morrumbene	3	14	1	1	1	0	42.86	4	6.79
Mozambique	Mossuril	3	22	0	0	0	0	4.81	5	6.99
Mozambique	Muanza	0	4	1	1	1	0	44.90	4	6.76
Mozambique	Nacala-Velha	4	15	0	0	0	0	0.00	5	6.86
Mozambique	Namacurra	7	19	1	1	1	0	46.94	5	7.84
Mozambique	Namapa - Erati	1	9	0	0	0	0	0.00	5	7.15
Mozambique	Nicoadala	8	30	1	1	0	0	25.77	4	6.59
Mozambique	Palma	0	9	0	0	0	0	6.00	3	5.32
Mozambique	Pebane	7	19	1	1	1	0	30.73	5	7.69
Mozambique	Pemba	1	12	0	0	0	0	0.00	5	6.97
Mozambique	Quissanga	1	15	0	0	0	0	0.00	3	4.96
Mozambique	Vilankulo	5	12	1	1	1	0	31.42	4	6.54
Mozambique	Xai-Xai	6	28	1	1	1	1	89.47	3	6.01
Mozambique	Zavala	0	4	1	1	1	1	100.00	5	8.75
Tanzania	Bagamoyo	0	9	1	1	1	0	37.41	3	5.69
Tanzania	Chake	0	0	0	0	0	0	15.38	2	4.32
Tanzania	Ilala	0	0	0	0	0	0	0.00	1	3.02
Tanzania	Kaskazini 'A'	0	2	0	0	0	0	0.00	2	4.17
Tanzania	Kaskazini 'B'	0	0	0	0	0	0	0.00	2	4.14
Tanzania	Kati	0	0	0	0	0	0	0.00	1	3.20
Tanzania	Kilwa	0	0	0	0	0	0	4.34	2	4.12

Country	District	Number of Events causing deaths	Number of Events	>20% coastline at higher exposure (1 = yes)	>25% coastline at higher exposure (1 = yes)	>30% coastline at higher exposure (1 = yes)	>50% coastline at higher exposure (1 = yes)	% coastline at higher exposure (Scenario 1)	SVI	IVCC (Scenario 1)
Tanzania	Kinondoni	1	2	0	0	0	0	10.53	1	3.47
Tanzania	Kusini	0	0	0	0	0	0	0.00	1	3.24
Tanzania	Lindi Rural	0	0	0	0	0	0	0.00	3	5.07
Tanzania	Lindi Urban	0	2	0	0	0	0	0.00	3	4.87
Tanzania	Mafia	0	1	0	0	0	0	2.45	3	5.02
Tanzania	Magharibi	0	0	1	1	0	0	26.00	1	3.72
Tanzania	Micheweni	0	0	0	0	0	0	8.61	4	6.24
Tanzania	Mkinga	0	0	0	0	0	0	3.60	3	5.23
Tanzania	Mkoani	0	0	0	0	0	0	11.30	2	4.31
Tanzania	Mkuranga	0	0	1	1	1	0	32.76	3	5.66
Tanzania	Mtwara Rural	0	4	0	0	0	0	0.00	4	5.91
Tanzania	Mtwara Urban	0	0	0	0	0	0	0.00	2	3.63
Tanzania	Muheza	0	0	0	0	0	0	0.00	2	4.39
Tanzania	Pangani	0	0	1	1	1	0	31.52	2	4.76
Tanzania	Rufiji	1	11	1	1	1	0	46.88	2	4.98
Tanzania	Tanga	1	3	0	0	0	0	0.74	1	3.06
Tanzania	Temeke	0	2	0	0	0	0	2.54	1	3.17
Tanzania	Wete	0	0	0	0	0	0	7.88	2	4.24
Tanzania	Zanzibar town	0	0	0	0	0	0	0.00	1	3.22

Table S4 notes:
-The data of number of events and number of events causing death were obtained from the United Nations Desinventar database (https://www.desinventar.net/).

-The social vulnerability index (SVI) and the index of vulnerability for coastal change (IVCC) were not calculated for Madagascar as the available Census data was outdated (a decade older) than the other countries not and considered not comparable.

Table S5. Mann-Whitney Test results comparing the number of events causing death and the number of events between exposed and not-exposed districts defined using different thresholds of the proportion of shoreline at higher exposure

Threshold	Districts	N	Mean Rank	Sum of Ranks	Mann-Whitney test
**Number of events causing death**					
≤20%	Not-Exposed	61	48.74	2973	U=1082
>20%	Exposed	59	72.66	4287	**p<0.000****
≤25%	Not-Exposed	64	50.33	3221	U=1141
>25%	Exposed	56	72.13	4039	**p<0.000****
≤30%	Not-Exposed	75	52.75	3956	U=1106
>30%	Exposed	45	73.42	3304	**p<0.001****
≤50%	Not-Exposed	93	55.26	5139	U=768
>50%	Exposed	27	78.56	2121	**p<0.002****
**Number of events**					
≤20%	Not-Exposed	61	50.98	3110	U=1219
>20%	Exposed	59	70.34	4150	**p=0.002****
≤25%	Not-Exposed	64	51.95	3324.5	U=1244.5
>25%	Exposed	56	70.28	3935.5	**p=0.004****
≤30%	Not-Exposed	75	53.31	3998.5	U=1148.5
>30%	Exposed	45	72.48	3261.5	**p=0.003****
≤50%	Not-Exposed	93	56.92	5294	U=923
>50%	Exposed	27	72.81	1966	**p=0.036***

** significant at the 0.01 level (2-tailed).

* significant at the 0.05 level (2-tailed).

Table S5 notes: All statistical analysis were performed in IBM SPSS version 26.

Table S6. Spearman's rho correlations between the number of events causing death, the number of events, SVI values and IVCC values at district level (county level for Kenya) and descriptive stadistics.

		deaths	events	Shore	SVI	IVCC
deaths	Correlation Coefficient	1.000	.725**	.408**	.404**	.497**
	Sig. (2-tailed)		0.000	0.000	0.000	0.000
	N	120	120	120	75	75
events	Correlation Coefficient	.725**	1.000	.321**	.584**	.647**
	Sig. (2-tailed)	0.000		0.000	0.000	0.000
	N	120	120	120	75	75
Shore	Correlation Coefficient	.408**	.321**	1.000	.236*	.444**
	Sig. (2-tailed)	0.000	0.000		0.042	0.000
	N	120	120	120	75	75
SVI	Correlation Coefficient	.404**	.584**	.236*	1.000	.964**
	Sig. (2-tailed)	0.000	0.000	0.042		0.000
	N	75	75	75	75	75
IVCC	Correlation Coefficient	.497**	.647**	.444**	.964**	1.000
	Sig. (2-tailed)	0.000	0.000	0.000	0.000	
	N	75	75	75	75	75

**. Correlation is significant at the 0.01 level (2-tailed).

*. Correlation is significant at the 0.05 level (2-tailed).

Descriptive Statistics

	Mean	Maximum	Std. Deviation	N
deaths	2.48	15	2.768	120
events	8.33	43	7.504	120
Shore	29.20	100	29.781	120
SVI	3.31	5	1.355	75
IVCC	5.76	8.75	1.476	75
