# Supplementary material for: Integrated Assessment of Coastal Exposure and Social Vulnerability to Coastal Hazards in East Africa
Source: Estuaries Coast. 2021 May 13;44(8):2056–72. doi: 10.1007/s12237-021-00930-5 (PMC8118621; doi:10.1007/s12237-021-00930-5)
Supplement: Supplementary file 3 — (PDF 893 kb) [file 12237_2021_930_MOESM3_ESM.pdf]

Electronic Supplementary Material 3. Data

Integrated assessment of coastal exposure and social vulnerability to coastal hazards in East Africa

Estuaries and Coasts

Dr Caridad Ballesteros, cballesteros@bournemouth.ac.uk

Dr Luciana S. Esteves, lesteves@bournemouth.ac.uk (corresponding author)

Department of Life & Environmental Sciences

Faculty of Science & Technology

Bournemouth University

Fern Barrow, Poole, BH12 5BB, UK

Table S7. Total shoreline length, proportion of total shoreline length at higher exposure levels and average of Index of Exposure (IE) per country, province and district for each habitat scenario

Administrative divisions	Total shoreline length (km)	% of total shoreline length at higher exposure					average IE				
		Scenario 1	Scenario 2	Scenario 3	Scenario 4	Scenario 5	Scenario 1	Scenario 2	Scenario 3	Scenario 4	Scenario 5
**Kenya**	**1591**	**14.3**	**24.6**	**20.7**	**14.3**	**36.5**	**2.3**	**2.4**	**2.6**	**2.3**	**2.8**
Lamu	874	10.1	14.8	18.0	10.1	27.9	2.2	2.3	2.5	2.2	2.7
Lamu East	510	5.9	11.2	14.5	5.9	28.6	2.2	2.3	2.5	2.2	2.7
Lamu West	364	15.9	19.8	22.8	15.9	26.9	2.3	2.4	2.6	2.3	2.8
Tana River	99	64.6	71.7	69.7	64.6	76.8	3.1	3.2	3.4	3.1	3.5
Garsen	99	64.6	71.7	69.7	64.6	76.8	3.1	3.2	3.4	3.1	3.5
Kilifi	267	22.1	45.3	27.7	22.1	51.3	2.5	2.7	2.7	2.5	3.0
Magarini	91	24.2	35.2	35.2	24.2	50.5	2.5	2.7	2.8	2.5	3.0
Malindi	15	33.3	46.7	33.3	33.3	46.7	2.6	2.9	2.6	2.6	2.9
Kilifi North	136	23.5	43.4	27.2	23.5	44.9	2.4	2.7	2.6	2.4	2.9
Kilifi South	25	0.0	92.0	0.0	0.0	92.0	2.7	3.2	2.7	2.7	3.2
Mombasa	113	0.0	16.8	0.0	0.0	19.5	2.0	2.1	2.2	2.0	2.4
Kisauni	13	0.0	38.5	0.0	0.0	38.5	2.2	2.5	2.3	2.2	2.6
Nyali	14	0.0	42.9	0.0	0.0	57.1	2.4	2.7	2.5	2.4	2.9
Jomvu	12	0.0	0.0	0.0	0.0	0.0	1.7	1.7	2.0	1.7	2.0
Changamwe	27	0.0	0.0	0.0	0.0	0.0	1.9	1.9	2.2	1.9	2.2
Mvita	18	0.0	0.0	0.0	0.0	5.6	1.8	1.9	2.0	1.8	2.2
Likoni	29	0.0	27.6	0.0	0.0	27.6	2.0	2.2	2.2	2.0	2.4
Kwale	238	7.1	21.8	12.6	7.1	42.9	2.3	2.5	2.5	2.3	2.8
Matuga	17	0.0	52.9	0.0	0.0	76.5	2.4	2.7	2.5	2.4	2.9
Msambweni	92	0.0	22.8	0.0	0.0	46.7	2.2	2.4	2.4	2.2	2.7
Lungalunga	129	13.2	17.1	23.3	13.2	35.7	2.3	2.5	2.6	2.3	2.9
**Tanzania**	**3138**	**9.8**	**19.3**	**15.3**	**9.8**	**33.1**	**2.3**	**2.4**	**2.4**	**2.3**	**2.8**
Tanga	380	9.2	21.1	20.3	9.2	38.4	2.3	2.4	2.5	2.3	2.8

Administrative divisions	Total shoreline length (km)	% of total shoreline length at higher exposure					average IE				
		Scenario 1	Scenario 2	Scenario 3	Scenario 4	Scenario 5	Scenario 1	Scenario 2	Scenario 3	Scenario 4	Scenario 5
Mkinga	139	3.6	13.7	17.3	3.6	28.8	2.2	2.3	2.5	2.2	2.7
Tanga	136	0.7	5.1	5.9	0.7	19.1	2.1	2.2	2.3	2.1	2.6
Muheza	13	0.0	23.1	0.0	0.0	46.2	2.4	2.7	2.5	2.4	2.9
Pangani	92	31.5	55.4	48.9	31.5	80.4	2.8	3.0	3.0	2.8	3.4
Pemba North	354	8.2	22.0	9.9	8.2	30.8	2.2	2.5	2.4	2.2	2.8
Micheweni	151	8.6	19.2	9.9	8.6	27.8	2.2	2.5	2.4	2.2	2.7
Wete	203	7.9	24.1	9.9	7.9	33.0	2.2	2.5	2.4	2.2	2.8
Pemba South	268	12.7	22.0	20.5	12.7	43.7	2.3	2.5	2.5	2.3	2.9
Chake	91	15.4	25.3	19.8	15.4	42.9	2.3	2.5	2.5	2.3	2.9
Mkoani	177	11.3	20.3	20.9	11.3	44.1	2.3	2.5	2.5	2.3	2.9
Pwani	636	25.0	34.1	35.8	25.0	53.3	2.5	2.6	2.7	2.5	3.0
Bagamoyo	147	37.4	44.2	46.9	37.4	68.0	2.7	2.9	3.0	2.7	3.4
Mkuranga	116	32.8	42.2	44.8	32.8	66.4	2.7	2.8	2.9	2.7	3.3
Mafia	245	2.4	11.4	3.3	2.4	17.6	2.0	2.2	2.1	2.0	2.5
Rufiji	128	46.9	58.6	77.3	46.9	93.0	3.0	3.1	3.4	3.0	3.6
Zanzibar N	149	0.0	9.4	0.0	0.0	10.1	2.2	2.5	2.2	2.2	2.6
Kaskazini 'A'	108	0.0	10.2	0.0	0.0	11.1	2.2	2.5	2.2	2.2	2.6
Kaskazini 'B'	41	0.0	7.3	0.0	0.0	7.3	2.1	2.4	2.2	2.1	2.6
Zanzibar S and C	176	0.0	13.1	1.1	0.0	15.9	2.2	2.5	2.3	2.2	2.7
Kati	103	0.0	7.8	1.9	0.0	12.6	2.2	2.4	2.4	2.2	2.6
Kusini	73	0.0	20.5	0.0	0.0	20.5	2.2	2.6	2.3	2.2	2.7
Zanzibar West	110	23.6	46.4	30.0	23.6	61.8	2.7	3.0	2.8	2.7	3.2
Magharibi	100	26.0	49.0	33.0	26.0	66.0	2.7	3.0	2.8	2.7	3.2
Mjini (Zanz.town)	10	0.0	20.0	0.0	0.0	20.0	2.2	2.5	2.3	2.2	2.7
Dar es Salaam	182	4.9	11.0	9.3	4.9	35.7	2.3	2.5	2.4	2.3	2.8
Kinondoni	57	10.5	21.1	14.0	10.5	57.9	2.5	2.8	2.6	2.5	3.1
Ilala	7	0.0	0.0	0.0	0.0	14.3	2.0	2.3	2.1	2.0	2.5

Administrative divisions	Total shoreline length (km)	% of total shoreline length at higher exposure					average IE				
		Scenario 1	Scenario 2	Scenario 3	Scenario 4	Scenario 5	Scenario 1	Scenario 2	Scenario 3	Scenario 4	Scenario 5
Temeke	118	2.5	6.8	7.6	2.5	26.3	2.2	2.4	2.3	2.2	2.7
Lindi	603	2.8	9.6	5.3	2.8	21.6	2.1	2.2	2.2	2.1	2.6
Kilwa	392	4.3	12.2	8.2	4.3	24.0	2.1	2.3	2.3	2.1	2.6
Lindi Rural	121	0.0	7.4	0.0	0.0	19.8	2.1	2.2	2.2	2.1	2.6
Lindi Urban	90	0.0	1.1	0.0	0.0	13.3	1.9	2.0	2.1	1.9	2.3
Mtwara	280	0.0	1.8	0.7	0.0	7.9	1.9	2.0	2.1	1.9	2.3
Mtwara Rural	247	0.0	2.0	0.8	0.0	8.9	1.9	2.0	2.1	1.9	2.3
Mtwara Urban	33	0.0	0.0	0.0	0.0	0.0	1.6	1.7	1.8	1.6	2.0
**Mozambique**	**7146**	**23.4**	**27.9**	**29.5**	**23.7**	**35.0**	**2.5**	**2.5**	**2.7**	**2.5**	**2.8**
Cabo Delgado	1237	5.3	22.8	7.4	5.3	26.8	2.2	2.5	2.3	2.2	2.6
Palma	384	6.0	28.6	6.0	6.0	28.6	2.3	2.6	2.3	2.3	2.6
Mocimboa da Praia	140	15.7	35.7	19.3	15.7	46.4	2.4	2.7	2.5	2.4	2.9
Macomia	344	4.7	23.5	9.6	4.7	31.4	2.3	2.5	2.5	2.3	2.7
Quissanga	118	0.0	7.6	0.0	0.0	9.3	2.0	2.1	2.2	2.0	2.3
Pemba	189	0.0	10.6	2.1	0.0	13.2	2.0	2.1	2.1	2.0	2.3
Ancuabe	21	0.0	0.0	0.0	0.0	0.0	1.8	2.2	1.8	1.8	2.2
Mecufi	34	11.8	35.3	11.8	11.8	35.3	2.4	2.6	2.4	2.4	2.8
Chiure	7	0.0	0.0	0.0	0.0	0.0	2.0	2.0	2.3	2.0	2.3
Nampula	1665	8.7	12.5	11.4	8.7	15.5	2.2	2.3	2.3	2.2	2.5
Namapa	50	0.0	14.0	0.0	0.0	14.0	2.1	2.4	2.3	2.1	2.6
Memba	200	0.0	1.0	0.0	0.0	1.0	2.0	2.2	2.0	2.0	2.3
Nacala Velha	113	0.0	0.0	0.0	0.0	0.0	1.9	2.1	1.9	1.9	2.2
Mossuril	416	4.8	9.9	7.0	4.8	13.2	2.0	2.1	2.1	2.0	2.3
Mongincual	162	11.7	25.3	22.2	11.7	35.8	2.2	2.3	2.5	2.2	2.5
Angoche	520	7.3	9.4	10.4	7.3	12.5	2.2	2.2	2.6	2.2	2.6
Moma	204	33.3	33.3	34.8	33.3	34.8	2.7	2.7	2.8	2.7	2.8
Zambezia	1289	32.2	32.2	41.7	32.2	41.7	2.7	2.7	3.0	2.7	3.0

Administrative divisions	Total shoreline length (km)	% of total shoreline length at higher exposure					average IE				
		Scenario 1	Scenario 2	Scenario 3	Scenario 4	Scenario 5	Scenario 1	Scenario 2	Scenario 3	Scenario 4	Scenario 5
Pebane	449	30.7	30.7	34.3	30.7	34.3	2.7	2.7	2.9	2.7	2.9
Maganja da Costa	210	52.4	52.4	57.6	52.4	57.6	3.1	3.1	3.3	3.1	3.3
Namacurra	49	46.9	46.9	65.3	46.9	65.3	2.8	2.8	3.2	2.8	3.2
Nicoadala	163	25.8	25.8	33.7	25.8	33.7	2.6	2.6	3.0	2.6	3.0
Inhassunge	211	11.4	11.4	20.4	11.4	20.4	2.2	2.2	2.6	2.2	2.6
Chinde	207	37.7	37.7	64.3	37.7	64.3	2.8	2.8	3.3	2.8	3.3
Sofala	1126	26.0	26.0	43.3	26.0	43.3	2.5	2.5	3.0	2.5	3.0
Marromeu	127	28.3	28.3	49.6	28.3	49.6	2.6	2.6	3.0	2.6	3.0
Cheringoma	62	38.7	38.7	59.7	38.7	59.7	2.7	2.7	3.1	2.7	3.1
Muanza	127	44.9	44.9	59.8	44.9	59.8	2.8	2.8	3.2	2.8	3.2
Dondo	102	63.7	63.7	76.5	63.7	76.5	3.1	3.1	3.6	3.1	3.6
Buzi	267	26.6	26.6	41.2	26.6	41.2	2.5	2.5	3.0	2.5	3.0
Machanga	441	9.1	9.1	27.9	9.1	27.9	2.3	2.3	2.7	2.3	2.7
Inhambane	1189	36.5	40.3	39.4	36.5	43.2	2.7	2.7	2.8	2.7	2.8
Govuro	247	18.6	18.6	30.0	18.6	30.0	2.4	2.4	2.8	2.4	2.8
Inhassoro	139	36.0	36.0	36.0	36.0	36.0	2.7	2.8	2.7	2.7	2.8
Vilanculos	261	31.4	33.0	31.4	31.4	33.0	2.5	2.6	2.6	2.5	2.6
Massinga	138	53.6	61.6	58.7	53.6	66.7	2.8	2.8	2.8	2.8	2.9
Morrumbene	63	42.9	55.6	42.9	42.9	55.6	2.8	2.9	2.8	2.8	2.9
Homoine	49	14.3	14.3	14.3	14.3	14.3	2.5	2.5	2.5	2.5	2.5
Jangamo	162	14.2	25.3	14.2	14.2	25.3	2.5	2.6	2.5	2.5	2.6
Inharrime	54	90.7	98.1	90.7	90.7	98.1	3.2	3.3	3.2	3.2	3.3
Zavala	76	100.0	100.0	100.0	100.0	100.0	3.7	3.7	3.7	3.7	3.7
Gaza	202	76.2	76.2	76.2	76.2	76.2	2.9	2.9	2.9	2.9	2.9
Mandlakazi	58	100.0	100.0	100.0	100.0	100.0	3.3	3.3	3.3	3.3	3.3
Xai-Xai	76	89.5	89.5	89.5	89.5	89.5	3.0	3.0	3.0	3.0	3.0
Bilene	68	41.2	41.2	41.2	41.2	41.2	2.5	2.5	2.5	2.5	2.5

Administrative divisions	Total shoreline length (km)	% of total shoreline length at higher exposure					average IE				
		Scenario 1	Scenario 2	Scenario 3	Scenario 4	Scenario 5	Scenario 1	Scenario 2	Scenario 3	Scenario 4	Scenario 5
Maputo	313	41.5	42.2	45.0	48.9	55.6	2.8	2.8	2.9	2.8	3.0
Boane	1	0.0	0.0	0.0	0.0	0.0	2.8	2.8	2.8	2.8	2.8
Manhica	21	100.0	100.0	100.0	100.0	100.0	3.2	3.2	3.2	3.2	3.2
Marracuene	121	52.9	52.9	60.3	52.9	60.3	3.0	3.0	3.3	3.0	3.3
Matutuine	170	26.5	27.6	27.6	40.0	47.1	2.6	2.6	2.6	2.6	2.7
Maputo City	125	27.2	27.2	32.8	28.8	35.2	2.7	2.7	2.8	2.7	2.8
Maputo	125	27.2	27.2	32.8	28.8	35.2	2.7	2.7	2.8	2.7	2.8
**Madagascar**	**10237**	**25.6**	**32.8**	**33.0**	**25.8**	**43.8**	**2.5**	**2.6**	**2.7**	**2.5**	**2.9**
Diana	2039	12.1	18.7	17.4	12.1	29.7	2.2	2.4	2.4	2.2	2.7
Ambanja	651	9.4	14.1	18.1	9.4	28.3	2.2	2.3	2.5	2.2	2.7
Ambilobe	299	43.8	55.2	55.2	43.8	66.6	2.8	2.9	3.1	2.8	3.3
Antsiranana Rural	910	5.5	11.8	7.4	5.5	20.9	2.0	2.2	2.1	2.0	2.5
Nosibe	179	2.2	10.1	2.8	2.2	17.9	2.2	2.4	2.3	2.2	2.7
Sava	639	22.7	39.1	23.8	22.7	43.2	2.5	2.7	2.5	2.5	2.8
Antalaha	297	18.5	51.2	18.5	18.5	51.2	2.5	2.9	2.5	2.5	2.9
Sambava	86	68.6	68.6	68.6	68.6	68.6	3.0	3.0	3.0	3.0	3.0
Vohimarina (Iharana)	256	12.1	15.2	14.8	12.1	25.4	2.2	2.3	2.3	2.2	2.5
Analanjirofo	761	39.7	58.7	39.7	39.7	58.7	2.8	3.1	2.8	2.8	3.1
Fenoarivo Atsinanana	70	67.1	90.0	67.1	67.1	90.0	3.2	3.5	3.2	3.2	3.5
Mananara	230	26.1	56.1	26.1	26.1	56.1	2.7	3.1	2.7	2.7	3.1
Maroansetra	186	38.2	50.5	38.2	38.2	50.5	2.9	3.0	2.9	2.9	3.0
Nosy-Boraha (St. Marie)	164	28.7	41.5	28.7	28.7	41.5	2.5	2.8	2.5	2.5	2.8
Soanierana-Ivongo	111	69.4	83.8	69.4	69.4	83.8	3.3	3.4	3.3	3.3	3.4
Atsinanana	491	74.5	76.8	74.5	74.5	76.8	3.3	3.3	3.3	3.3	3.3

Administrative divisions	Total shoreline length (km)	% of total shoreline length at higher exposure					average IE				
		Scenario 1	Scenario 2	Scenario 3	Scenario 4	Scenario 5	Scenario 1	Scenario 2	Scenario 3	Scenario 4	Scenario 5
Ampasimanolotra	94	100.0	100.0	100.0	100.0	100.0	3.7	3.7	3.7	3.7	3.7
Mahanoro	195	50.8	50.8	50.8	50.8	50.8	2.9	2.9	2.9	2.9	2.9
Toamasina Rural	128	82.8	91.4	82.8	82.8	91.4	3.4	3.5	3.4	3.4	3.5
Vatomandry	74	90.5	90.5	90.5	90.5	90.5	3.5	3.6	3.5	3.5	3.6
Atsimo-Atsinana	278	75.2	75.2	75.2	75.2	75.2	3.2	3.2	3.2	3.2	3.2
Farafangana	88	100.0	100.0	100.0	100.0	100.0	3.7	3.7	3.7	3.7	3.7
Vangaindrano	190	63.7	63.7	63.7	63.7	63.7	3.0	3.0	3.0	3.0	3.0
Vatovavy Fitovinany	405	70.4	71.1	70.4	70.4	71.1	3.3	3.4	3.3	3.3	3.4
Manakara-Sud	123	60.2	62.6	60.2	60.2	62.6	3.1	3.3	3.1	3.1	3.3
Mananjary	147	77.6	77.6	77.6	77.6	77.6	3.5	3.5	3.5	3.5	3.5
Nosy varika	95	67.4	67.4	67.4	67.4	67.4	3.2	3.2	3.2	3.2	3.2
Vohipeno	40	82.5	82.5	82.5	82.5	82.5	3.4	3.4	3.4	3.4	3.4
Anosy	408	42.4	49.5	42.4	43.1	51.5	2.9	3.0	2.9	2.9	3.0
Amboasary-Sud	27	81.5	81.5	81.5	81.5	81.5	3.2	3.2	3.2	3.2	3.2
Taolagnaro	381	39.6	47.2	39.6	40.4	49.3	2.9	3.0	2.9	2.9	3.0
Androy	211	81.5	85.8	81.5	84.4	88.6	3.2	3.2	3.2	3.2	3.2
Ambovombe-Androy	55	90.9	90.9	90.9	90.9	90.9	3.2	3.2	3.2	3.2	3.2
Beloha	70	65.7	78.6	65.7	74.3	87.1	3.0	3.1	3.0	3.0	3.1
Tsiombe	86	88.4	88.4	88.4	88.4	88.4	3.3	3.3	3.3	3.3	3.3
Atsimo-Andrefana	927	9.2	22.0	15.4	9.7	35.6	2.4	2.6	2.5	2.4	2.8
Ampanihy	238	15.5	31.9	15.5	15.5	31.9	2.6	2.8	2.6	2.6	2.8
Betioky-Sud	99	5.1	12.1	5.1	5.1	13.1	2.3	2.7	2.3	2.3	2.8
Morombe	411	9.0	13.9	21.7	10.2	34.8	2.3	2.4	2.5	2.3	2.7
Toliary	155	3.2	36.1	6.5	3.2	54.8	2.4	2.7	2.5	2.4	2.9
Toliary Urban	24	4.2	12.5	8.3	4.2	54.2	2.4	2.6	2.5	2.4	3.0
Menabe	854	22.4	25.9	39.0	22.4	42.3	2.5	2.5	2.9	2.5	2.9

Administrative divisions	Total shoreline length (km)	% of total shoreline length at higher exposure					average IE				
		Scenario 1	Scenario 2	Scenario 3	Scenario 4	Scenario 5	Scenario 1	Scenario 2	Scenario 3	Scenario 4	Scenario 5
Belon-i Tsiribihina	349	20.3	20.3	40.1	20.3	40.1	2.4	2.4	2.9	2.4	2.9
Manja	149	28.2	33.6	45.6	28.2	51.0	2.6	2.7	3.0	2.6	3.1
Morondava	356	21.9	28.1	35.1	21.9	40.7	2.4	2.5	2.8	2.4	2.9
Melaky	871	32.3	32.6	49.9	32.3	49.9	2.6	2.6	3.0	2.6	3.0
Antsalova	162	29.6	29.6	43.8	29.6	43.8	2.6	2.6	3.0	2.6	3.0
Besalampy	259	40.9	40.9	59.5	40.9	59.5	2.8	2.8	3.2	2.8	3.2
Maintirano	450	28.2	28.9	46.7	28.2	46.7	2.5	2.5	3.0	2.5	3.0
Boeny	1171	10.2	14.8	23.4	10.6	33.6	2.3	2.4	2.7	2.3	2.8
Mahajanga Rural	468	4.9	5.8	14.5	5.8	24.6	2.3	2.3	2.6	2.3	2.7
Mahajanga Urban	28	14.3	42.9	21.4	14.3	50.0	2.6	2.7	2.7	2.6	2.9
Mitsinjo	396	10.4	14.6	26.0	10.4	34.3	2.3	2.4	2.7	2.3	2.8
Soalala	279	18.6	27.2	34.8	18.6	46.2	2.4	2.5	2.8	2.4	2.9
Sofia	1182	3.8	12.1	15.0	3.8	30.8	2.3	2.4	2.6	2.3	2.7
Analalava	1066	3.6	12.8	15.1	3.6	32.2	2.3	2.4	2.6	2.3	2.8
Antsihihy	29	0.0	0.0	0.0	0.0	0.0	2.1	2.1	2.5	2.1	2.5
Port Bergé	87	8.0	8.0	18.4	8.0	24.1	2.2	2.2	2.5	2.2	2.6

Table S7 note:

Habitat scenarios

Scenario 1 all habitats
Scenario 2 no corals
Scenario 3 no mangroves
Scenario 4 no seagrasses
Scenario 5 no habitats

Table S8. Social Vulnerability Index (SVI), average of Index of Exposure (IE) at the district level for each habitat scenarios, Index of Vulnerability to Coastal Change (IVCC) for each habitat scenarios, people exposed (within 5km) to higher levels of exposure for scenarios 1,2,3 and 5

Country	Province /County	District	SVI	average IE					IVCC					Population exposed			
				S. 1	S. 2	S. 3	S. 4	S. 5	S. 1	S. 2	S. 3	S. 4	S.5	S. 1	S. 2	S. 3	S. 5
KEN	Lamu	Lamu East	3	2.2	2.3	2.5	2.2	2.7	5.2	5.3	5.5	5.2	5.7	3,774	4,472	4,690	5,490
		Lamu West	3	2.3	2.4	2.6	2.3	2.8	5.3	5.4	5.6	5.3	5.8	41,475	43,994	45,611	45,709
	Tana River	Garsen	5	3.1	3.2	3.4	3.1	3.5	8.1	8.2	8.4	8.1	8.5	15,159	15,360	15,426	15,626
	Kilifi	Magarini	5	2.5	2.7	2.8	2.5	3.0	7.5	7.7	7.8	7.5	8.0	45,566	47,036	48,685	49,386
		Malindi	2	2.6	2.9	2.6	2.6	2.9	4.6	4.9	4.6	4.6	4.9	110,198	110,975	110,198	110,975
		Kilifi North	3	2.4	2.7	2.6	2.4	2.9	5.4	5.7	5.6	5.4	5.9	82,468	164,769	85,776	165,532
		Kilifi South	3	2.7	3.2	2.7	2.7	3.2	5.7	6.2	5.7	5.7	6.2		96,277		96,277
	Mombasa	Kisauni	2	2.2	2.5	2.3	2.2	2.6	4.2	4.5	4.3	4.2	4.6		147,559		147,559
		Nyali	1	2.4	2.7	2.5	2.4	2.9	3.4	3.7	3.5	3.4	3.9		215,878		215,878
		Jomvu	2	1.7	1.7	2.0	1.7	2.0	3.7	3.7	4.0	3.7	4.0				
		Changamwe	1	1.9	1.9	2.2	1.9	2.2	2.9	2.9	3.2	2.9	3.2				
		Mvita	1	1.8	1.9	2.0	1.8	2.2	2.8	2.9	3.0	2.8	3.2		99,368		127,371
		Likoni	1	2.0	2.2	2.2	2.0	2.4	3.0	3.2	3.2	3.0	3.4		142,770		143,913
	Kwale	Matuga	4	2.4	2.7	2.5	2.4	2.9	6.4	6.7	6.5	6.4	6.9		38,885		38,885
		Msambweni	2	2.2	2.4	2.4	2.2	2.7	4.2	4.4	4.4	4.2	4.7		108,136		112,451
		Lungalunga	5	2.3	2.5	2.6	2.3	2.9	7.3	7.5	7.6	7.3	7.9	15,987	17,212	18,777	23,452
TNZ	Tanga	Mkinga	3	2.2	2.3	2.5	2.2	2.7	5.2	5.3	5.5	5.2	5.7	4,165	6,558	13,654	13,885
		Tanga	1	2.1	2.2	2.3	2.1	2.6	3.1	3.2	3.3	3.1	3.6	733	3,815	58,415	137,884
		Muheza	2	2.4	2.7	2.5	2.4	2.9	4.4	4.7	4.5	4.4	4.9	382	2,320	382	2,613
		Pangani	2	2.8	3.0	3.0	2.8	3.4	4.8	5.0	5.0	4.8	5.4	7,081	16,495	15,708	18,111
	Pemba North	Micheweni	4	2.2	2.5	2.4	2.2	2.7	6.2	6.5	6.4	6.2	6.7	34,366	41,817	40,222	75,300
		Wete	2	2.2	2.5	2.4	2.2	2.8	4.2	4.5	4.4	4.2	4.8	18,777	26,657	20,067	28,596
	Pemba South	Chake	2	2.3	2.5	2.5	2.3	2.9	4.3	4.5	4.5	4.3	4.9	29,777	31,875	31,354	36,978
		Mkoani	2	2.3	2.5	2.5	2.3	2.9	4.3	4.5	4.5	4.3	4.9	51,881	53,643	55,311	67,860
	Pwani	Bagamoyo	3	2.7	2.9	3.0	2.7	3.4	5.7	5.9	6.0	5.7	6.4	33,491	39,859	35,541	41,357

Country	Province /County	District	SVI	average IE S. 1	average IE S. 2	average IE S. 3	average IE S. 4	average IE S. 5	IVCC S. 1	IVCC S. 2	IVCC S. 3	IVCC S. 4	IVCC S.5	Population exposed S. 1	Population exposed S. 2	Population exposed S. 3	Population exposed S. 5
		Mkuranga	3	2.7	2.8	2.9	2.7	3.3	5.7	5.8	5.9	5.7	6.3	8,047	9,081	9,179	9,600
		Mafia	3	2.0	2.2	2.1	2.0	2.5	5.0	5.2	5.1	5.0	5.5	87	11,381	1,382	15,238
		Rufiji	2	3.0	3.1	3.4	3.0	3.6	5.0	5.1	5.4	5.0	5.6	11,957	13,022	14,468	14,621
	Zanzibar N	Kaskazini 'A'	2	2.2	2.5	2.2	2.2	2.6	4.2	4.5	4.2	4.2	4.6		41,623		42,188
		Kaskazini 'B'	2	2.1	2.4	2.2	2.1	2.6	4.1	4.4	4.2	4.1	4.6		7,370		7,370
	Zanzibar S and C	Kati	1	2.2	2.4	2.4	2.2	2.6	3.2	3.4	3.4	3.2	3.6	2,955	13,874	10,538	22,108
		Kusini	1	2.2	2.6	2.3	2.2	2.7	3.2	3.6	3.3	3.2	3.7		24,032		25,547
	Zanzibar W	Magharibi	1	2.7	3.0	2.8	2.7	3.2	3.7	4.0	3.8	3.7	4.2	27,200	180,403	29,589	205,690
		Mjini (Stone Town)	1	2.2	2.5	2.3	2.2	2.7	3.2	3.5	3.3	3.2	3.7	4,577	245,098	4,577	245,098
	Dar es Salaam	Kinondoni	1	2.5	2.8	2.6	2.5	3.1	3.5	3.8	3.6	3.5	4.1	162,211	573,046	182,745	780,596
		Ilala	1	2.0	2.3	2.1	2.0	2.5	3.0	3.3	3.1	3.0	3.5		110,098		197,680
		Temeke	1	2.2	2.4	2.3	2.2	2.7	3.2	3.4	3.3	3.2	3.7	1,731	95,621	2,709	164,092
	Lindi	Kilwa	2	2.1	2.3	2.3	2.1	2.6	4.1	4.3	4.3	4.1	4.6	5,305	16,607	11,295	24,188
		Lindi Rural	3	2.1	2.2	2.2	2.1	2.6	5.1	5.2	5.2	5.1	5.6		3,991		7,294
		Lindi Urban	3	1.9	2.0	2.1	1.9	2.3	4.9	5.0	5.1	4.9	5.3		497		3,205
	Mtwara	Mtwara Rural	4	1.9	2.0	2.1	1.9	2.3	5.9	6.0	6.1	5.9	6.3	326	7,845	3,189	17,609
		Mtwara Urban	2	1.6	1.7	1.8	1.6	2.0	3.6	3.7	3.8	3.6	4.0				
MOZ	Cabo Delgado	Palma	3	2.3	2.6	2.3	2.3	2.6	5.3	5.6	5.3	5.3	5.6	2,845	8,193	2,845	8,193
		Mocimboa da Praia	4	2.4	2.7	2.5	2.4	2.9	6.4	6.7	6.5	6.4	6.9	2,573	7,738	4,626	8,837
		Macomia	4	2.3	2.5	2.5	2.3	2.7	6.3	6.5	6.5	6.3	6.7	6,591	11,140	10,178	17,307
		Quissanga	3	2.0	2.1	2.2	2.0	2.3	5.0	5.1	5.2	5.0	5.3		509		591
		Pemba	5	2.0	2.1	2.1	2.0	2.3	7.0	7.1	7.1	7.0	7.3		95,906	3,735	98,583
		Ancuabe	5	1.8	2.2	1.8	1.8	2.2	6.8	7.2	6.8	6.8	7.2				
		Mecufi	4	2.4	2.6	2.4	2.4	2.8	6.4	6.6	6.4	6.4	6.8	3,487	5,253	3,487	5,253
		Chiúre	4	2.0	2.0	2.3	2.0	2.3	6.0	6.0	6.3	6.0	6.3	242	242	242	242
	Nampula	Namapa	5	2.1	2.4	2.3	2.1	2.6	7.1	7.4	7.3	7.1	7.6		1,314		1,314
		Memba	5	2.0	2.2	2.0	2.0	2.3	7.0	7.2	7.0	7.0	7.3		4,144		4,144
		Nacala Velha	5	1.9	2.1	1.9	1.9	2.2	6.9	7.1	6.9	6.9	7.2		3,363		3,363

Country	Province /County	District	SVI	average IE					IVCC					Population exposed			
				S. 1	S. 2	S. 3	S. 4	S. 5	S. 1	S. 2	S. 3	S. 4	S.5	S. 1	S. 2	S. 3	S. 5
		Mossuril	5	2.0	2.1	2.1	2.0	2.3	7.0	7.1	7.1	7.0	7.3	14,436	45,615	16,654	48,010
		Mongincual	5	2.2	2.3	2.5	2.2	2.5	7.2	7.3	7.5	7.2	7.5	14,410	19,917	15,975	21,453
		Angoche	5	2.2	2.2	2.6	2.2	2.6	7.2	7.2	7.6	7.2	7.6	39,162	43,354	48,405	51,787
		Moma	5	2.7	2.7	2.8	2.7	2.8	7.7	7.7	7.8	7.7	7.8	28,255	28,255	28,255	28,255
		Pebane	5	2.7	2.7	2.9	2.7	2.9	7.7	7.7	7.9	7.7	7.9	18,995	18,995	19,808	19,808
		Maganja da Costa	4	3.1	3.1	3.3	3.1	3.3	7.1	7.1	7.3	7.1	7.3	32,644	32,644	34,332	34,332
	Zambezia	Namacurra	5	2.8	2.8	3.2	2.8	3.2	7.8	7.8	8.2	7.8	8.2	16,401	16,401	19,999	19,999
		Nicoadala	4	2.6	2.6	3.0	2.6	3.0	6.6	6.6	7.0	6.6	7.0	38,959	38,959	60,636	60,636
		Inhassunge	4	2.2	2.2	2.6	2.2	2.6	6.2	6.2	6.6	6.2	6.6	18,674	18,674	23,388	23,388
		Chinde	4	2.8	2.8	3.3	2.8	3.3	6.8	6.8	7.3	6.8	7.3	18,383	18,383	19,707	19,707
		Marromeu	4	2.6	2.6	3.0	2.6	3.0	6.6	6.6	7.0	6.6	7.0	6,953	6,953	7,749	7,749
		Cheringoma	5	2.7	2.7	3.1	2.7	3.1	7.7	7.7	8.1	7.7	8.1	979	979	1,099	1,099
	Sofala	Muanza	4	2.8	2.8	3.2	2.8	3.2	6.8	6.8	7.2	6.8	7.2	1,607	1,607	1,667	1,667
		Dondo	2	3.1	3.1	3.6	3.1	3.6	5.1	5.1	5.6	5.1	5.6	150,530	150,530	151,109	151,109
		Buzi	4	2.5	2.5	3.0	2.5	3.0	6.5	6.5	7.0	6.5	7.0	7,436	7,436	7,786	7,786
		Machanga	4	2.3	2.3	2.7	2.3	2.7	6.3	6.3	6.7	6.3	6.7	9,500	9,500	12,652	12,652
		Govuro	4	2.4	2.4	2.8	2.4	2.8	6.4	6.4	6.8	6.4	6.8	2,133	2,133	2,758	2,758
		Inhassoro	3	2.7	2.8	2.7	2.7	2.8	5.7	5.8	5.7	5.7	5.8	3,697	3,697	3,697	3,697
		Vilanculos	4	2.5	2.6	2.6	2.5	2.6	6.5	6.6	6.6	6.5	6.6	18,411	18,988	18,411	18,988
		Massinga	3	2.8	2.8	2.8	2.8	2.9	5.8	5.8	5.8	5.8	5.9	22,254	23,208	22,441	23,339
	Inhambane	Morrumbene	4	2.8	2.9	2.8	2.8	2.9	6.8	6.9	6.8	6.8	6.9	15,809	16,807	15,809	16,807
		Homoine	5	2.5	2.5	2.5	2.5	2.5	7.5	7.5	7.5	7.5	7.5	24,456	24,456	24,456	24,456
		Jangamo	5	2.5	2.6	2.5	2.5	2.6	7.5	7.6	7.5	7.5	7.6	42,701	49,993	42,701	49,993
		Inharrime	5	3.2	3.3	3.2	3.2	3.3	8.2	8.3	8.2	8.2	8.3	17,244	17,445	17,244	17,445
		Zavala	5	3.7	3.7	3.7	3.7	3.7	8.7	8.7	8.7	8.7	8.7	24,975	24,975	24,975	24,975
		Mandlakazi	4	3.3	3.3	3.3	3.3	3.3	7.3	7.3	7.3	7.3	7.3	19,597	19,597	19,597	19,597
	Gaza	Xai-Xai	3	3.0	3.0	3.0	3.0	3.0	6.0	6.0	6.0	6.0	6.0	53,018	53,018	53,018	53,018
		Bilene	3	2.5	2.5	2.5	2.5	2.5	5.5	5.5	5.5	5.5	5.5	3,716	3,716	3,716	3,716

Country	Province /County	District	SVI	average IE S. 1	S. 2	S. 3	S. 4	S. 5	IVCC S. 1	S. 2	S. 3	S. 4	S.5	Population exposed S. 1	S. 2	S. 3	S. 5
	Maputo	Boane	3	2.8	2.8	2.8	2.8	2.8	5.8	5.8	5.8	5.8	5.8	491	491	491	491
		Manhiça	5	3.2	3.2	3.2	3.2	3.2	8.2	8.2	8.2	8.2	8.2	1,781	1,781	1,781	1,781
		Marracuene	3	3.0	3.0	3.3	3.0	3.3	6.0	6.0	6.3	6.0	6.3	43,667	43,667	44,199	44,199
		Matutuine	2	2.6	2.6	2.6	2.6	2.7	4.6	4.6	4.6	4.6	4.7	3,919	4,290	3,996	5,779
	Maputo City	Maputo	1	2.7	2.7	2.8	2.7	2.8	3.7	3.7	3.8	3.7	3.8	929,458	929,458	929,458	931,575
MAD	Diana	Ambanja		2.2	2.3	2.5	2.2	2.7						28,469	33,340	33,330	39,729
		Ambilobe		2.8	2.9	3.1	2.8	3.3						28,579	28,579	31,177	31,177
		Antsiranana Rural		2.0	2.2	2.1	2.0	2.5						5,844	13,903	8,501	33,589
		Nosibe		2.2	2.4	2.3	2.2	2.7						942	45,319	4,511	61,818
	Sava	Antalaha		2.5	2.9	2.5	2.5	2.9						19,739	47,845	19,739	47,845
		Sambava		3.0	3.0	3.0	3.0	3.0						33,640	33,640	33,640	33,640
		Vohimarina (Iharana)		2.2	2.3	2.3	2.2	2.5						12,288	13,365	26,848	29,643
	Analanjirofo	Fenoarivo Atsinanana		3.2	3.5	3.2	3.2	3.5						45,418	46,890	45,418	46,890
		Mananara		2.7	3.1	2.7	2.7	3.1						22,020	28,460	22,020	28,460
		Maroansetra		2.9	3.0	2.9	2.9	3.0						19,069	23,974	19,069	23,974
		Nosy-Boraha (St. Marie)		2.5	2.8	2.5	2.5	2.8						25,259	27,896	25,259	27,896
		Soanierana-Ivongo		3.3	3.4	3.3	3.3	3.4						15,611	16,043	15,611	16,043
	Atsinanana	Ampasimanolotra		3.7	3.7	3.7	3.7	3.7						8,330	8,330	8,330	8,330
		Mahanoro		2.9	2.9	2.9	2.9	2.9						26,125	26,125	26,125	26,125
		Toamasina Rural		3.4	3.5	3.4	3.4	3.5						165,675	165,772	165,675	165,772
		Vatomandry		3.5	3.6	3.5	3.5	3.6						24,623	24,623	24,623	24,623
	Atsimo-Atsinana	Farafangana		3.7	3.7	3.7	3.7	3.7						104,539	104,539	104,539	104,539
		Vangaindrano		3.0	3.0	3.0	3.0	3.0						45,016	45,016	45,016	45,016
		Manakara-Sud		3.1	3.3	3.1	3.1	3.3						83,545	84,737	83,545	84,737

Country	Province /County	District	SVI	average IE					IVCC					Population exposed			
				S. 1	S. 2	S. 3	S. 4	S. 5	S. 1	S. 2	S. 3	S. 4	S.5	S. 1	S. 2	S. 3	S. 5
	Vatovavy Fitovinany	Mananjary		3.5	3.5	3.5	3.5	3.5						45,828	45,828	45,828	45,828
		Nosy varika		3.2	3.2	3.2	3.2	3.2						20,946	20,946	20,946	20,946
		Vohipeno		3.4	3.4	3.4	3.4	3.4						24,612	24,612	24,612	24,612
	Anosy	Amboasary-Sud		3.2	3.2	3.2	3.2	3.2						9,851	9,851	9,851	9,851
		Taolagnaro		2.9	3.0	2.9	2.9	3.0						56,649	60,354	56,649	60,644
	Androy	Ambovombe-Androy		3.2	3.2	3.2	3.2	3.2						47,043	47,043	47,043	47,043
		Beloha		3.0	3.1	3.0	3.0	3.1						5,615	5,838	5,615	5,890
		Tsiombe		3.3	3.3	3.3	3.3	3.3						19,190	19,190	19,190	19,190
	Atsimo-Andrefana	Ampanihy		2.6	2.8	2.6	2.6	2.8						4,459	11,864	4,459	11,864
		Betioky-Sud		2.3	2.7	2.3	2.3	2.8						1,339	2,695	1,339	2,945
		Morombe		2.3	2.4	2.5	2.3	2.7						5,089	8,091	11,607	13,267
		Toliary		2.4	2.7	2.5	2.4	2.9						3,670	14,215	5,859	32,608
		Toliary Urban		2.4	2.6	2.5	2.4	3.0						8,638	16,753	13,183	28,648
	Menabe	Belon-i Tsiribihina		2.4	2.4	2.9	2.4	2.9						8,427	8,427	10,301	10,301
		Manja		2.6	2.7	3.0	2.6	3.1						1,857	1,862	2,051	2,057
		Morondava		2.4	2.5	2.8	2.4	2.9						46,128	46,194	47,356	47,454
	Melaky	Antsalova		2.6	2.6	3.0	2.6	3.0						3,785	3,785	3,987	3,987
		Besalampy		2.8	2.8	3.2	2.8	3.2						5,374	5,374	5,678	5,678
		Maintirano		2.5	2.5	3.0	2.5	3.0						20,841	21,542	22,773	22,773
	Boeny	Mahajanga Rural		2.3	2.3	2.6	2.3	2.7						69,216	100,308	121,989	124,824
		Mahajanga Urban		2.6	2.7	2.7	2.6	2.9						15,051	17,981	30,855	31,428
		Mitsinjo		2.3	2.4	2.7	2.3	2.8						4,060	4,868	8,979	9,252
		Soalala		2.4	2.5	2.8	2.4	2.9						4,022	4,407	6,087	6,369
	Sofia	Analalava		2.3	2.4	2.6	2.3	2.8						9,160	15,933	21,411	26,567
		Antsihihy		2.1	2.1	2.5	2.1	2.5								90	90
		Port Bergé		2.2	2.2	2.5	2.2	2.6						1,194	1,194	1,629	1,740

Table S9. Average of Social Vulnerability Index (SVI), average of Index of Exposure (IE) at the province and country level for each habitat scenarios, Index of Vulnerability to Coastal Change (IVCC) for each habitat scenarios, people exposed (within 5km) to higher levels of exposure for scenarios 1,2,3 and 5.

Administrative divisions	SVI	average IE					IVCC					Population exposed			
		S. 1	S. 2	S. 3	S. 4	S. 5	S. 1	S. 2	S. 3	S. 4	S. 5	S. 1	S. 2	S. 3	S. 5
**Kenya**	**2.6**	**2.3**	**2.4**	**2.6**	**2.3**	**2.8**	**4.9**	**5.0**	**5.2**	**4.9**	**5.4**	**314,627**	**1,252,693**	**329,163**	**1,298,504**
Lamu	3.0	2.2	2.3	2.5	2.2	2.7	5.2	5.3	5.5	5.2	5.7	45,249	48,466	50,301	51,199
Tana River	5.0	3.1	3.2	3.4	3.1	3.5	8.1	8.2	8.4	8.1	8.5	15,159	15,360	15,426	15,626
Kilifi	3.3	2.5	2.7	2.7	2.5	3.0	5.7	6.0	5.9	5.7	6.2	238,233	419,058	244,660	422,170
Mombasa	1.3	2.0	2.1	2.2	2.0	2.4	3.3	3.4	3.5	3.3	3.7		605,576		634,721
Kwale	3.6	2.3	2.5	2.5	2.3	2.8	5.9	6.1	6.1	5.9	6.4	15,987	164,233	18,777	174,787
**Tanzania**	**2.0**	**2.3**	**2.4**	**2.4**	**2.3**	**2.8**	**4.3**	**4.4**	**4.4**	**4.3**	**4.8**	**405,050**	**1,576,628**	**540,325**	**2,204,709**
Tanga	2.0	2.3	2.4	2.5	2.3	2.8	4.3	4.4	4.5	4.3	4.8	12,361	29,188	88,159	172,493
Pemba N	3.0	2.2	2.5	2.4	2.2	2.8	5.2	5.5	5.4	5.2	5.8	53,144	68,474	60,289	103,895
Pemba S	2.0	2.3	2.5	2.5	2.3	2.9	4.3	4.5	4.5	4.3	4.9	81,658	85,518	86,665	104,839
Pwani	2.8	2.5	2.6	2.7	2.5	3.0	5.2	5.4	5.5	5.2	5.8	53,582	73,344	60,570	80,816
Zanzibar N	2.0	2.2	2.5	2.2	2.2	2.6	4.2	4.5	4.2	4.2	4.6		48,993		49,558
Zanzibar W	1.0	2.7	3.0	2.8	2.7	3.2	3.7	4.0	3.8	3.7	4.2	31,778	425,501	34,166	450,788
Zanzibar S & C	1.0	2.2	2.5	2.3	2.2	2.7	3.2	3.5	3.3	3.2	3.7	2,955	37,906	10,538	47,655
Dar es Salaam	1.0	2.3	2.5	2.4	2.3	2.8	3.3	3.5	3.4	3.3	3.8	163,942	778,765	185,453	1,142,368
Lindi	2.6	2.1	2.2	2.2	2.1	2.6	4.7	4.8	4.8	4.7	5.2	5,305	21,095	11,295	34,686
Mtwara	3.0	1.9	2.0	2.1	1.9	2.3	4.9	5.0	5.1	4.9	5.3	326	7,845	3,189	17,609
**Mozambique**	**3.5**	**2.5**	**2.5**	**2.7**	**2.5**	**2.8**	**6.0**	**6.0**	**6.2**	**6.0**	**6.3**	**1,660,391**	**1,833,723**	**1,723,082**	**1,899,877**
Cabo Delgado	3.7	2.2	2.5	2.3	2.2	2.6	5.9	6.2	6.0	5.9	6.3	15,737	128,982	25,112	139,005
Nampula	4.6	2.2	2.3	2.3	2.2	2.5	6.8	6.9	6.9	6.8	7.1	96,264	145,961	109,289	158,326
Zambezia	4.0	2.7	2.7	3.0	2.7	3.0	6.7	6.7	7.0	6.7	7.0	144,056	144,056	177,869	177,869
Sofala	3.4	2.5	2.5	3.0	2.5	3.0	5.9	5.9	6.4	5.9	6.4	177,004	177,004	182,063	182,063
Inhambane	4.2	2.7	2.7	2.8	2.7	2.8	6.9	6.9	7.0	6.9	7.0	171,681	181,701	172,492	182,457
Gaza	2.7	2.9	2.9	2.9	2.9	2.9	5.6	5.6	5.6	5.6	5.6	76,331	76,331	76,331	76,331
Maputo	3.2	2.8	2.8	2.9	2.8	3.0	6.0	6.0	6.1	6.0	6.2	49,858	50,229	50,467	52,251

Administrative divisions	SVI	average IE					IVCC					Population exposed			
		**S. 1**	**S. 2**	**S. 3**	**S. 4**	**S. 5**	**S. 1**	**S. 2**	**S. 3**	**S. 4**	**S. 5**	**S. 1**	**S. 2**	**S. 3**	**S. 5**
Maputo City	1.2	2.7	2.7	2.8	2.7	2.8	3.9	3.9	4.0	3.9	4.0	929,458	929,458	929,458	931,575
**Madagascar**		**2.5**	**2.6**	**2.7**	**2.5**	**2.9**						**1,156,774**	**1,337,553**	**1,292,344**	**1,495,702**
Analanjirofo		2.8	3.1	2.8	2.8	3.1						127,376	143,263	127,376	143,263
Androy		3.2	3.2	3.2	3.2	3.2						71,847	72,070	71,847	72,123
Anosy		2.9	3.0	2.9	2.9	3.0						66,500	70,205	66,500	70,495
Atsimo-Andrefana		2.4	2.6	2.5	2.4	2.8						23,195	53,619	36,447	89,332
Atsimo-Atsinana		3.2	3.2	3.2	3.2	3.2						149,556	149,556	149,556	149,556
Atsinanana		3.3	3.3	3.3	3.3	3.3						224,754	224,851	224,754	224,851
Boeny		2.3	2.4	2.7	2.3	2.8						92,348	127,564	167,909	171,873
Diana		2.2	2.4	2.4	2.2	2.7						63,834	121,140	77,520	166,313
Melaky		2.6	2.6	3.0	2.6	3.0						30,000	30,701	32,438	32,438
Menabe		2.5	2.5	2.9	2.5	2.9						56,412	56,483	59,709	59,812
Sava		2.5	2.7	2.5	2.5	2.8						65,667	94,849	80,227	111,127
Sofia		2.3	2.4	2.6	2.3	2.7						10,354	17,127	23,130	28,397
Vatovavy Fitovinany		3.3	3.4	3.3	3.3	3.4						174,932	176,124	174,932	176,124

Table S8 and S9 note:

Habitat scenarios

S. 1: Scenario 1 all habitats
S. 2: Scenario 2 no corals
S. 3: Scenario 3 no mangroves
S. 4: Scenario 4 no seagrasses
S. 5: Scenario 5 no habitats

Table S10. Social Vulnerability Index (SVI) indicators

Country	Province/ County	District	% <4 years old	% >65 years old	% Floor Material	% Wall Material	% Roof Material	% Average House Material	% Unimproved sources of water	% Unimproved human waste disposal	% Pop. growth	Pop. density (Person/ km^2)	% Illiterate rate
KEN	Lamu	Lamu East	18.1	4.1	56.0	43.3	90.8	63.4	82.6	28.1	3.6	7.4	43.5
		Lamu West	18.9	3.3	64.3	58.9	84.4	69.2	39.0	46.8	3.6	19.3	30.3
	Tana River	Garsen	22.8	3.1	91.9	84.5	98.7	91.7	45.6	79.7	3.6	6.1	51.0
	Kilifi	Magarini	22.6	3.6	84.6	80.0	95.7	86.8	70.9	83.0	3.6	25.3	39.9
		Malindi	19.0	2.8	42.9	38.4	88.8	56.7	12.6	44.3	3.6	263.5	29.3
		Kilifi North	20.0	3.2	62.2	53.5	96.3	70.7	9.8	54.3	3.6	245.9	31.6
		Kilifi South	19.5	3.1	57.1	46.0	93.7	65.6	22.7	31.4	3.6	392.2	29.6
	Mombasa	Kisauni	17.4	1.3	21.8	15.6	88.7	42.0	24.7	21.0	3.6	1758.4	19.0
		Nyali	15.7	1.5	13.6	10.3	87.0	37.0	25.9	19.7	3.6	7336.4	14.9
		Jomvu	17.5	1.3	32.4	33.0	90.8	52.1	17.5	20.6	3.6	2671.3	18.2
		Changamwe	15.6	1.2	20.0	22.6	88.3	43.6	38.5	12.8	3.6	6256.0	13.0
		Mvita	12.3	3.6	9.9	10.7	45.3	22.0	21.7	9.2	3.6	8617.4	18.2
		Likoni	17.4	1.2	13.8	8.0	94.3	38.7	17.5	24.0	3.6	2990.0	18.1
	Kwale	Matuga	20.1	3.5	70.3	59.8	98.0	76.0	44.7	53.3	2.7	157.7	35.1
		Msambweni	18.0	2.7	40.5	31.6	96.2	56.1	25.6	54.0	2.7	285.9	27.1
		Lungalunga	22.9	3.5	86.2	77.8	98.2	87.4	75.8	83.5	2.7	75.6	45.0
TAN	Tanga	Mkinga	14.9	4.8	75.1	77.2	97.8	83.4	63.6	80.5	2.1	42.2	26.2
		Tanga	14.9	4.8	26.4	24.9	96.6	49.3	6.1	25.7	1.2	427.1	7.4
		Muheza	14.9	4.8	64.5	52.9	98.1	71.8	48.2	66.0	1.1	136.4	14.3
		Pangani	14.9	4.8	66.2	68.5	94.0	76.2	36.4	63.3	2.1	30.5	18.2
	Pemba N	Micheweni	17.6	3.1	68.0	56.3	99.2	74.5	40.6	67.8	2.2	407.2	43.9
		Wete	17.6	3.1	43.0	57.2	98.2	66.1	7.4	43.2	0.6	353.5	21.8
	Pemba S	Chake	17.1	3.3	45.5	63.4	98.9	69.3	13.7	41.2	1.6	431.2	24.1

Country	Province/ County	District	% <4 years old	% >65 years old	% Floor Material	% Wall Material	% Roof Material	% Average House Material	% Unimproved sources of water	% Unimproved human waste disposal	% Pop. growth	Pop. density (Person/ km^2)	% Iliterate rate
		Mkoani	17.1	3.3	51.8	75.6	98.9	75.4	17.3	51.4	0.6	364.8	29.4
	Pwani	Bagamoyo	14.0	6.2	60.0	67.2	99.8	75.7	43.7	69.3	3.1	36.8	24.6
		Mkuranga	14.0	6.2	66.8	70.8	99.8	79.1	60.4	72.3	1.8	79.0	32.6
		Mafia	14.0	6.2	59.8	78.9	99.5	79.4	58.5	69.7	1.4	97.6	22.7
		Rufiji	14.0	6.2	80.4	83.2	99.7	87.8	60.1	82.9	0.7	16.0	36.7
	Zanzibar N	Kaskazini 'A'	19.8	2.6	44.6	13.7	98.9	52.4	23.3	32.8	2.3	444.5	23.4
		Kaskazini 'B'	19.8	2.6	44.0	30.9	97.8	57.6	14.0	44.0	4.4	344.4	16.4
	Zanzibar S and C	Kati	14.3	3.7	34.4	24.0	97.0	51.8	43.1	22.6	2.0	147.7	12.7
		Kusini	14.3	3.7	29.1	10.9	94.0	44.7	7.4	13.9	2.1	102.9	9.1
	Zanzibar W	Magharibi	14.6	2.1	10.6	5.2	98.8	38.2	9.6	8.8	7.0	1612.9	7.3
		Mjini (Stone Town)	14.6	2.1	3.3	0.4	93.8	32.5	4.9	4.3	0.8	13531.6	6.3
	Dar es Salaam	Kinondoni	12.1	2.1	2.8	4.6	98.2	35.2	29.9	11.0	4.9	3299.3	2.4
		Ilala	12.1	2.1	2.2	4.9	94.1	33.7	19.7	10.2	6.5	3353.3	2.9
		Temeke	12.1	2.1	2.4	4.9	97.9	35.1	11.4	11.0	5.8	1865.0	4.6
	Lindi	Kilwa	13.0	6.3	81.6	85.1	99.7	88.8	68.4	81.5	1.1	12.5	35.4
		Lindi Rural	13.0	6.3	91.4	82.0	99.5	91.0	57.0	92.1	0.6	118.3	39.7
		Lindi Urban	13.0	6.3	58.5	50.3	98.8	69.2	46.4	60.7	2.5	149.2	27.3
	Mtwara	Mtwara Rural	13.3	6.7	91.0	88.2	99.6	92.9	67.0	92.4	0.9	259.8	42.2
		Mtwara Urban	13.3	6.7	45.4	45.4	97.2	62.7	8.4	51.0	2.4	1788.4	16.6
MOZ	Cabo Delgado	Palma	15.7	3.9	95.2	99.0	100.0	98.1	67.3	97.4	0.7	14.4	71.5
		Mocimboa da Praia	14.5	4.6	90.3	96.3	99.7	95.4	64.7	91.3	2.1	28.8	63.8

Country	Province/ County	District	% <4 years old	% >65 years old	% Floor Material	% Wall Material	% Roof Material	% Average House Material	% Unimproved sources of water	% Unimproved human waste disposal	% Pop. growth	Pop. density (Person/ km^2)	% Iliterate rate
		Macomia	15.8	4.7	93.2	98.1	99.8	97.0	46.7	97.9	1.5	20.5	68.0
		Quissanga	18.0	4.2	94.7	98.8	99.9	97.8	71.3	97.4	0.6	18.6	67.2
		Pemba	17.2	3.7	96.2	98.8	99.9	98.3	41.5	93.1	3.1	46.8	64.8
		Cidade de Pemba	12.9	1.9	60.8	83.0	95.8	79.9	4.6	55.4	4.5	1711.5	28.2
		Ancuabe	18.9	3.4	97.6	99.2	99.8	98.9	74.6	95.5	1.3	23.6	69.7
		Mecufi	18.8	4.5	95.2	99.0	99.9	98.0	16.6	95.0	1.2	37.6	70.9
		Chiúre	18.2	2.5	97.9	99.1	100.0	99.0	79.8	95.3	1.5	43.8	72.7
	Nampula	Namapa	18.1	2.9	98.1	98.5	100.0	98.9	91.7	98.0	2.1	50.5	75.6
		Memba	18.3	3.2	97.9	99.3	99.9	99.0	91.3	97.7	1.4	56.1	75.3
		Nacala Velha	15.7	4.2	95.5	98.7	100.0	98.1	73.2	95.7	3.5	92.6	73.7
		Cidade de Nacala	15.2	2.5	69.4	85.8	97.5	84.2	27.8	72.2	1.6	713.3	47.6
		Mossuril	17.1	5.6	94.5	97.8	99.8	97.4	80.1	97.4	1.8	38.3	74.3
		Ilha de Mocambique (Cidade)	15.7	4.5	70.6	82.3	97.9	83.6	42.1	88.6	1.4	287.7	52.5
		Mongincual	17.4	3.1	97.6	99.2	99.9	98.9	79.4	96.3	2.6	34.1	72.2
		Angoche	16.8	3.9	91.4	94.1	98.3	94.6	70.1	95.8	1.8	101.8	67.2
		Moma	16.5	3.1	96.6	98.9	99.7	98.4	81.9	98.5	1.8	60.2	66.6
		Pebane	18.2	3.0	97.0	99.1	99.9	98.7	77.7	98.8	2.3	20.7	63.8
	Zambezia	Maganja da Costa	18.2	2.5	98.0	99.0	99.9	99.0	79.2	98.9	1.5	39.3	74.0
		Namacurra	16.0	2.8	97.1	98.2	99.9	98.4	70.5	98.5	4.9	113.1	64.0
		Nicoadala	16.2	3.2	96.1	97.0	99.9	97.7	79.6	97.5	1.3	73.8	56.6

Country	Province/ County	District	% <4 years old	% >65 years old	% Floor Material	% Wall Material	% Roof Material	% Average House Material	% Unimproved sources of water	% Unimproved human waste disposal	% Pop. growth	Pop. density (Person/ km^2)	% Iliterate rate
		Cidade de Quelimane	11.4	1.9	71.5	85.5	97.3	84.8	11.4	55.8	2.6	1841.4	22.8
		Inhassunge	15.7	3.8	97.3	98.8	100.0	98.7	62.3	98.4	1.2	129.3	66.5
		Chinde	17.6	3.2	96.7	97.2	99.3	97.7	72.9	97.9	1.5	30.5	64.1
	Sofala	Marromeu	18.5	1.9	93.4	93.4	99.2	95.3	52.0	92.5	4.3	26.1	51.3
		Cheringoma	19.0	1.9	89.3	89.2	99.6	92.7	61.0	93.8	7.7	7.0	54.4
		Muanza	20.6	1.5	96.4	98.1	99.8	98.1	93.5	96.4	4.5	4.3	58.9
		Dondo	14.3	2.4	70.8	83.3	99.5	84.5	30.7	79.7	2.3	70.1	29.0
		Cidade da Beira	12.7	2.0	19.8	43.8	90.4	51.3	17.2	43.0	0.4	723.0	16.3
		Buzi	16.7	3.2	90.6	93.8	99.9	94.8	64.1	92.4	2.0	24.9	54.1
		Machanga	16.0	4.5	91.3	95.0	99.9	95.4	75.1	95.7	2.1	10.3	60.3
	Inhambane	Govuro	16.5	4.4	85.1	89.4	99.8	91.4	74.6	85.5	2.0	9.9	52.0
		Inhassoro	15.5	4.5	79.2	91.0	99.9	90.0	31.1	90.2	2.6	11.7	49.8
		Vilanculos	15.6	4.8	75.0	89.9	99.8	88.2	57.7	89.3	2.0	26.5	46.2
		Massinga	16.7	4.8	61.9	79.0	99.2	80.0	55.0	83.8	0.8	26.9	42.8
		Morrumbe.	16.3	5.8	61.5	80.1	99.2	80.3	58.9	81.6	1.8	54.4	38.3
		Homoine	16.9	6.0	65.2	83.2	99.7	82.7	86.3	88.8	1.8	63.4	39.2
		Jangamo	17.7	5.5	63.2	80.7	99.0	81.0	67.2	89.9	1.7	81.2	40.9
		Inharrime	18.0	5.0	73.0	86.9	99.2	86.4	75.8	89.5	2.9	42.3	41.0
		Zavala	18.0	5.7	65.3	78.8	99.8	81.3	60.0	87.5	1.4	76.4	36.3
	Gaza	Mandlakazi	16.7	6.2	61.7	79.0	99.6	80.1	58.5	86.6	1.2	47.7	30.4
		Xai-Xai	16.6	4.1	44.9	77.4	99.8	74.0	41.1	76.5	2.3	126.6	32.2
		Cidade de Xai-Xai	13.1	2.5	14.2	68.8	96.9	60.0	10.1	34.1	1.3	918.8	14.4

Country	Province/ County	District	% <4 years old	% >65 years old	% Floor Material	% Wall Material	% Roof Material	% Average House Material	% Unimproved sources of water	% Unimproved human waste disposal	% Pop. growth	Pop. density (Person/ km^2)	% Iliterate rate
		Bilene	16.2	4.2	40.3	71.3	99.5	70.4	45.3	83.0	1.4	74.9	29.6
		Boane	15.8	3.5	33.9	36.7	96.9	55.8	19.5	66.9	4.9	166.7	26.8
	Maputo	Manhiça	14.6	4.4	48.1	70.9	98.8	72.6	41.6	81.4	6.6	119.4	38.4
		Marracuene	16.2	3.4	25.8	48.7	98.5	57.7	37.0	60.9	6.3	170.7	22.6
		Matutuine	15.9	5.5	30.8	83.2	97.9	70.6	58.8	83.8	1.0	7.5	42.2
		Cidade De Matola	11.7	2.0	10.6	16.7	93.3	40.2	14.9	39.4	3.9	2252.2	11.6
		Kalhamanku.	10.9	3.4	4.3	8.3	87.1	33.2	0.1	23.2	0.3	22674.7	7.5
		Kamavota	11.9	2.1	5.2	8.7	97.2	37.0	6.4	16.9	1.9	5148.6	8.6
	Maputo City	Kamaxakeni	11.6	2.6	4.1	6.9	98.1	36.4	0.7	16.5	0.3	7710.5	8.5
		Kamubukwa.	12.0	2.1	6.4	14.2	94.3	38.3	12.5	23.2	2.5	4959.6	7.0
		Kampfumu	7.8	2.8	0.3	1.5	12.2	4.7	0.1	1.1	0.2	7369.2	1.4
		Katembe	12.4	5.3	28.4	44.9	94.1	55.8	14.5	53.2	1.4	217.1	14.8
		Kanyaka	13.4	5.2	35.5	78.5	99.5	71.2	85.7	89.6	0.7	114.8	18.4
